# PAK3 activation promotes the tangential to radial migration switch of cortical interneurons by increasing leading process dynamics and disrupting cell polarity

**DOI:** 10.1038/s41380-024-02483-y

**Published:** 2024-03-07

**Authors:** Lucie Viou, Melody Atkins, Véronique Rousseau, Pierre Launay, Justine Masson, Clarisse Pace, Fujio Murakami, Jean-Vianney Barnier, Christine Métin

**Affiliations:** 1 INSERM UMR-S 1270; Institut du Fer à Moulin, Sorbonne Université, F-75005 Paris, France; 2 Institut des Neurosciences Paris-Saclay, UMR 9197, CNRS, Université Paris-Saclay, Saclay, France; 3 Graduate School of Frontier Biosciences, Osaka University, Yamadaoka 1-3, Suita, Osaka 565-0871 Japan

**Keywords:** Neuroscience, Cell biology

## Abstract

Mutations of PAK3, a p21-activated kinase, are associated in humans with cognitive deficits suggestive of defective cortical circuits and with frequent brain structural abnormalities. Most human variants no longer exhibit kinase activity. Since GABAergic interneurons express *PAK3* as they migrate within the cortex, we here examined the role of PAK3 kinase activity in the regulation of cortical interneuron migration. During the embryonic development, cortical interneurons migrate a long distance tangentially and then re-orient radially to settle in the cortical plate, where they contribute to cortical circuits. We showed that interneurons expressing a constitutively kinase active PAK3 variant (PAK3-ca) extended shorter leading processes and exhibited unstable polarity. In the upper cortical layers, they entered the cortical plate and extended radially oriented processes. In the deep cortical layers, they exhibited erratic non-processive migration movements and accumulated in the deep pathway. Pharmacological inhibition of PAK3 kinase inhibited the radial migration switch of interneurons to the cortical plate and reduced their accumulation in the deep cortical layers. Interneurons expressing a kinase dead PAK3 variant (PAK3-kd) developed branched leading processes, maintained the same polarity during migration and exhibited processive and tangentially oriented movements in the cortex. These results reveal that PAK3 kinase activity, by promoting leading process shortening and cell polarity changes, inhibits the tangential processive migration of interneurons and favors their radial re- orientation and targeting to the cortical plate. They suggest that patients expressing PAK3 variants with impaired kinase activity likely present alterations in the cortical targeting of their GABAergic interneurons.

## Introduction

Mutations in *PAK3*, which encodes a p21-activated kinase, are responsible in humans for intellectual disability, developmental delay including motor and speech deficits, behavioral abnormalities belonging to the autistic spectrum, aggression and violence, schizophreniform psychosis, severe epilepsy [1–6] and brain structural alterations [5–7]. PAK3 belongs to the group I PAK kinases, which are highly conserved in vertebrates with close structural and biochemical properties and a similar mechanism of activation by the RAC and CDC42 GTPases [8–11]. In group I PAKs, the binding of the activated (GTP linked) RAC or CDC42 GTPases dissociates PAK dimers and activates the PAK kinase domain. PAKs are thus important effectors of the small GTPases RAC or CDC42, even if adaptors proteins, lipids and numerous interacting proteins can moreover activate them. As a consequence of the RAC1 or CDC42 cycling activity, PAK proteins are cyclically activated and switch between inactive and activated conformations in physiological conditions [8]. RAC or CDC42 are known to regulate dynamic changes in the acto-myosin cytoskeleton, in microtubules and in adhesive interactions with the microenvironment [12–15]. Accordingly, PAK kinases play a central role in a large number of signaling cascades that regulate cell adhesion, cell motility and cell morphology in neurons and other cell types, and thereby contribute to the regulation of complex cellular mechanisms such as cell migration, neurite growth and dendritic spine morphogenesis [16–20] (see also reviews [8–11]).

Two members of the group I PAKs, PAK1 and PAK3 are highly expressed in the developing and adult brain [21–27] (see also reviews [8–10]). In the developing forebrain, PAK1 regulates the polarization, morphology and motility of the excitatory cortical neurons as they migrate along the radial glia [18, 24]. Importantly, PAK3 expression is restricted to neural cells and has been observed in migrating interneurons (INs), as early as embryonic day 11.5 (E11.5) [23].

Given the early expression of PAK3 in migrating inhibitory GABAergic INs, it is hypothesized that *PAK3* mutations could impair the development of inhibitory cortical circuits. This hypothesis is supported by observations that neuro-psychiatric diseases including epilepsy, schizophrenia, autism, can be correlated with abnormal location, density and ratios of IN subpopulations that control the firing of excitatory neurons in cortical circuits [28]. The role of PAK3 in the development of inhibitory circuits has been addressed in mice models that overexpress *Pak3* in INs, following the ablation of *Dlx1/2* transcription factors that repress *Pak3* expression in IN precursors [23, 26]. Cortical INs are born in *Dlx1/2* expressing domains, the medial and caudal ganglionic eminences (MGE, CGE) and the pre-optic area of the basal telencephalon. They reach the cortex during the embryonic development (reviewed in [29–31]). Within the embryonic cortex, INs first undergo a phase of tangential migration in two main migratory streams, a large one in the deep cortical layers, the intermediate zone and subventricular zone (iz/svz), and a superficial one at the brain surface in the marginal zone (mz). Then, INs switch from a tangential to a radial orientation to enter the cortical plate (cp) where they settle and differentiate [32–34]. This trajectory re-orientation of INs is operated in response to extrinsic guidance cues by changes of motility and polarity and by highly coordinated cytoskeletal and adhesion reorganizations [35–37].

Owing that the large majority of *PAK3* point mutations identified in human families target the kinase domain of the protein and lead to the expression of kinase inactive variants [6, 7], we here examined how the PAK3 kinase activity controls the migration of INs. We therefore transfected INs with PAK3 mutants locked either in a kinase activated (constitutively active PAK3-ca) or a kinase inactivated (kinase dead PAK3-kd) conformation and examined their migratory behavior. We first assessed the cellular properties of transfected cortical INs that migrate in a co-culture model designed to analyze the leading process morphology and the dynamics of growth cones, nucleus and centrosome. We next examined the directionality of INs expressing PAK3 mutants in co-cultures, and their cortical trajectories in organotypic forebrain slices. Finally, we examined the cortical distribution of transfected cortical INs ex vivo in cultured forebrain slices and in vivo, using in utero electroporation.

We showed here that cortical INs expressing a PAK3 mutant with a constitutive kinase activity no longer migrate tangentially but reorient their trajectories radially to the upper cortical layers. On the contrary, the lack of PAK3 kinase activity maintains INs in tangentially oriented pathways in the deep cortical layers. Our work strongly suggests that the expression of PAK3 pathogenic kinase variants in humans could thus affect brain circuit formation by disorganizing the cortical distribution of INs.

## Materials and methods

### Mice

Experiments have been validated and approved by the Ethical committee Charles Darwin (C2EA-05, authorized project 02241.02) and mice were housed and mated according to European guidelines. Embryos were produced by crosses between wild type (*Swiss*, Janvier, France) and/or genetically modified adults: CAG-mRFP1 transgenic mice [38], Myosin 2B-GFP mutant mice [39], Nkx2.1-cre,Rosa26-tdTomato transgenic mice (see supplementary Materials and methods). The day of vaginal plug was noted E0.5.

### Plasmids

pCAG-eGFP was a generous gift from Prof Miyzaki (Osaka University, Japan), pCAG-mRFP from Prof F. Murakami (Osaka University, Japan) and pCAG-PACT-mKO1 (pericentrin marker) from Prof F. Matsuzaki (RIKEN Center for Developmental Biology, Japan). They were used at a final concentration 0.5 µg/µL for in vitro and in utero electroporation. Construction of eGFP-PAK3 and mRFP-PAK3 plasmids used in the present study: see the Supplementary Methods. Before electroporation, constructs were diluted in PBS with 0.01% Fast Green at a final concentration of 1 µg/µL. For in utero electroporation, constructs were diluted at 1.5 µg/µL.

#### In vitro kinase assay

pCAG-GFP-PAK3 plasmids were co-transfected or not with active forms of Cdc42 or Rac1 GTPases in HEK cells. PAK3 proteins were immuno-precipitated and after normalization, immuno-purified proteins were tested in kinase assay.

### Cultures and in vitro electroporation

Brains and MGE explants of mouse embryos were collected and dissected at embryonic day E13.5 in cold Leibovitz medium (Invitrogen). MGE explants were electroporated as explained in [40] (and see Supplementary Methods). Electroporated explants were divided into smaller pieces and placed either on dissociated cortical cells or grafted into organotypic slices, either at the pallium-subpallium boundary or in the MGE. Slices were prepared from E14.5 embryonic brains embedded in 3% type VII agar (Sigma) and sectioned coronally using a manual slicer into 150 or 200 µm-thick sections. Slices were thereafter transferred in Millicell chambers (Merck Millipore) and cultured in the culture medium of co-cultures (see Supplementary Methods) supplemented with 10% fetal calf serum. Slices were transferred in a petri dish equipped with a glass coverslip before imaging. One hour before imaging, the culture medium was replaced by a culture medium of the same composition but without phenol red.

### Pharmacological treatment

FRAX597 [41] (Tocris), a group I PAK inhibitor, was added at 1 μM to the culture medium of grafted slices and renewed after 12 h. The stock solution was 10 mM in DMSO and the culture medium of control experiments contained DMSO 1/10,000 (vehicle).

### Videomicroscopy

#### Co-cultures

Time-lapse imaging was performed with an inverted microscope equipped with a spinning disk (Leica DMI4000) and with a temperature-controlled chamber. Cells were observed using either a ×20 objective (trajectories and morphological analyses), or ×40 or ×63 immersion objectives (subcellular analyses). Stacks were captured with a Coolsnap HQ camera (Roper Scientific, USA) every 3 or 5 min for at least 20 h for trajectory and morphology analyses, and every 1 min for myosin2B-GFP analyses. Control and mutant MGE cells were recorded at the same time in different sectors of the same co-culture.

#### Organotypic slices cultures

Time-lapse imaging of grafted organotypic slices cultured for 24 h was performed with an epifluorescence macroscope (MVX10 Olympus) equipped with a temperature-controlled chamber at 34–35 °C using a ×2×3.2 (or a ×2×4) objective. Pictures were acquired with a time interval of 5 or 10 min for at least 21 h. Acquisitions were controlled using the Metamorph software (Roper Scientific, USA).

### In utero electroporation

Ganglionic eminence directed in utero electroporation was performed as described in [42] on E12.5 swiss embryos. See supplementary information.

### Immunocytochemistry

Primary antibodies were chicken anti-GFP (1:5000 Aves Lab), rabbit anti-GFP serum (1:1000 Invitrogen), rabbit anti-RFP (1:1000, BioVision), rabbit anti-phosphorylated PAK3 (phospho-Ser154) (1:100, antibodies.com, A94421). Primary antibodies were revealed by immunofluorescence with the appropriate Alexa dye (Molecular Probes) or Cy3 (Jackson laboratories) conjugated secondary antibodies diluted in PBT (1:400). Bisbenzimide (1/5000 in PBT, Sigma) was used for nuclear counterstaining. Cultures were mounted in Mowiol-Dabco, organotypic slices in glycerol/PBS (2/1 vol/vol).

### Microscopy and image processing

Cultures were observed with a LEICA DM6000 upright fluorescent microscope. Grafted slices and in utero electroporated brain sections were imaged with a confocal microscope (Leica TCS SP5) using x10 or x40 objectives. Cells were counted and leading process orientation measured on Z-projection of confocal planes acquired every 2 microns in grafted cortical slices or sections of electroporated brains.

Cell counting in a volume or surface, leading process length measurement were performed with the Cell Counter and NeuronJ plugins of ImageJ (NIH, USA). Leading process orientation was measured with regards to the cortical surface as explained in [40]. Cell trajectories were reconstructed on movies by manually tracking the cell rear with MTrakJ (ImageJ plugin, NIH, USA) or with the tracking function of the Metamorph software. Dynamic parameters were extracted from tracking data.

To calculate the fluorescence index in Fig. 4D5, we measured the fluorescence intensity with the plot profile function of ImageJ under a 150 pixel-wide line and extracted peak values in the mz and grafted region.

### Statistical analyses

In text and figures, the variability around mean values is represented by s.e.m. (standard error of mean). Statistical analyses were performed with GraphPad Prism or R software using Student’s *T* tests (Mann–Whitney test for non Gaussian distributions), chi2 tests, One Way ANOVA (Kruskal-Wallis test for non Gaussian distribution) or Two-way ANOVA tests followed by Post-hoc tests (Bonferroni or Dunn) and Poisson-ANOVA models followed by likehood ratio tests, across n individuals, where n is the number of embryos or cells as specified in the figure legend. Values of *p* < 0.05 were considered significant. In figures, levels of significance were expressed by * for *p* < 0.05, ** for *p* < 0.01, *** for *p* < 0.001 and **** for *p* < 0.0001. Exact p values for *, **, *** are shown in legends.

## Results

### The expression of PAK3 mutants alters the morphology of migrating MGE cells

**Fig. 1:**
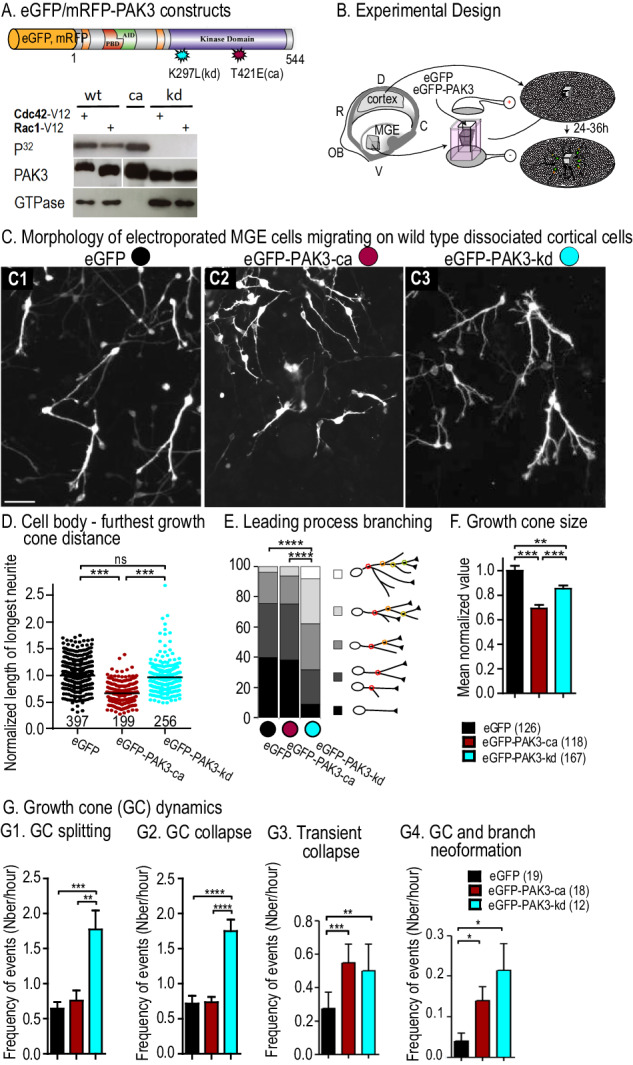
Leading process morphology and growth cone dynamics in MGE cells expressing PAK3 mutants. **A** Scheme of the electroporated murine PAK3 constructs (see Results). In vitro kinase assay shows that the PAK3-wt protein is activated by active GTPases, whereas the PAK3-ca protein displays high constitutive kinase activity and the PAK3-kd protein is totally devoid of activity. First row, auto-phosphorylation of mutants; second row, purified PAK3 mutants; third row, expression of GTPases in transfected cells. **B** To prepare co-cultures, MGE explants were dissected out of telencephalic vesicles at E13.5, electroporated and placed on a substrate of E13.5 wild type dissociated cortical cells. MGE cells migrated centrifugally away from MGE explants on the substrate of cortical cells. Leading process morphology was analyzed in fixed co-cultures (**C**–**E**). Dynamic transformations of growth cones were analyzed by time-lapse video-microscopy in living co-cultures (**F**, **G**). **C** Pictures show MGE cells expressing control eGFP (C1, black dot), eGFP-PAK3-ca (C2, red dot) and eGFP-PAK3-kd (C3, blue dot) constructs at the migration front. Scale bar, 20 μm. **D** Graph of the length of the longest neurite in large samples of electroporated cells (numbers below graphs). Lengths were normalized to the mean value in the control sample (eGFP expressing cells). Statistical significance was tested by the Kruskal–Wallis test (*p* < 0.0001) and Dunn’s post-hoc tests. **E** Histogram showing the percentage of MGE cells exhibiting a leading process without (black), with 1 (dark grey), 2 (medium grey), 3 (light grey), 4 or more (white) bifurcations (chi2 tests). **F** Histogram showing the mean surface (pixel number) of growth cones at their maximum size on movies. Values were normalized with regards to the mean growth cone size in MGE cells expressing eGFP (One Way ANOVA, *p* < 0.0001, and Bonferroni’s post-hoc tests for pair comparisons). **G** Histograms of the mean frequency of leading growth cone splitting (G1), collapse (G2), transient collapse (G3) and growth cone neoformation leading to a novel branch (G4) in MGE cells expressing eGFP (19 cells), eGFP-PAK3-ca (18 cells), and eGFP-PAK3-kd (12 cells) constructs analyzed for more than 2 h. In G1 and G2, statistical significance is assessed by the Kruskal-Wallis test (*p* = 0.0004 in H1, *P* < 0.0001 in G2) and Dunn’s post-hoc tests. In G3 and G4, statistical significance is assessed by the Poisson-ANOVA model, and post-hoc likehood ratio test, ***p = 0.0007, **p = 0.0031 in G3, and *p = 0.036, *p* = 0.0116 in G4. See also Supplementary Fig. S1A and Supplementary movie S1A.

To investigate the specific influence of the PAK3 kinase activity on IN migration, we inserted the murine PAK3 coding sequence fused to either eGFP or mRFP in a pCAG expression vector. Point mutations were introduced in the kinase domain of the wild type protein (PAK3-wt) to either generate a constitutively active kinase (« ca », T421E) or to abrogate the kinase activity (kinase dead « kd », K297L) [43] (Fig. 1A, see Supplementary Methods). We verified the constitutive activity and loss of kinase activity of the PAK3 constructs used in the present study by an in vitro kinase assay (Fig. 1A). Hereafter, PAK3 constructs fused to eGFP will be referred to as PAK3-wt, PAK3-ca, PAK3-kd. We analyzed the morphology of transfected MGE cells in co-cultures previously characterized in our laboratory [44], in which control MGE cells migrate actively away from their explant of origin on a flat substrate of dissociated cortical cells (Fig. 1B). Cell morphology and migration was analyzed at the front of migration in fixed co-cultures and by live cell imaging.

MGE cells expressing PAK3-ca had significantly shorter leading processes than MGE cells expressing eGFP and PAK3-kd constructs (Fig. 1C, D). The leading processes of MGE cells expressing PAK3-kd were significantly more branched than those of control and PAK3-ca expressing MGE cells (Fig. 1C, E). The growth cones at the tip of active neurites were significantly smaller in MGE expressing PAK3-ca than in MGE cells expressing eGFP and PAK3-kd (Fig. 1F). In control MGE cells, the leading growth cone regularly divided to produce paired branches (Fig. S1A, black dot and Movie S1A). One growth cone of the pair thereafter collapsed and the associated branch was eliminated. Transient collapse at the neurite tip followed by neuritic regrowth was sometimes observed and growth cones rarely neoformed along the leading process (Fig. 1G1–4, black bars). MGE cells expressing PAK3-ca showed the same frequency of growth cone splitting and collapse, but exhibited more frequent transient growth cone collapses than control cells, in agreement with their shorter leading processes (Fig. S1A, red dot, and Fig. 1G1-3, red bars). By comparison, MGE cells expressing PAK3-kd, which have highly branched leading processes, exhibited twice as much growth cone splitting as control cells, more frequent growth cone neoformation and –as expected- more frequent collapse and transient collapse than control MGE cells (Fig. S1A, blue dot and Fig. 1G1–4, blue bars).

Interestingly, the overexpression of the wild type protein (PAK3-wt) only weakly altered the morphology of migrating MGE cells (Fig. S1B), suggesting a preponderant role of PAK3 kinase activity rather than PAK3 expression level on the morphology of INs.

### Centrosomal and nuclear movements are altered in MGE cells expressing PAK3 mutants

**Fig. 2:**
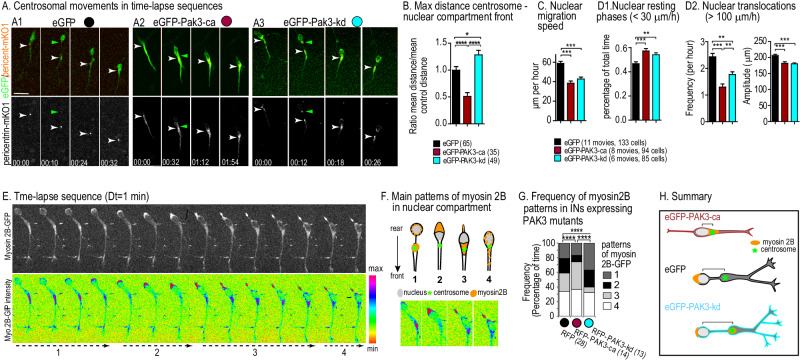
Abnormal centrosomal and nuclear movements in MGE cells expressing PAK3 mutants. **A** Time-lapse sequences illustrate the migration of MGE cells co-electroporated with eGFP (A1), eGFP-PAK3-ca (A2), eGFP-PAK3-kd (A3) and pericentrin-mKO1 plasmids. Pericentrin-mKO1 labels the centrosome (white in lower frames, white arrowheads). The nucleus is the oval structure at cell rear. On time-lapse sequences, we identified nuclear translocations and the forward centrosomal migration preceding each nuclear translocation. Time in hours:minutes on frames. Scale bar, 10 µm. **B** Histograms compare the maximal centrosomal-nuclear distance in time-lapse sequences (shown on frames with green and white arrowheads in A). Statistical significance is assessed by the One Way ANOVA test (*p* < 0.0001) and Bonferroni’s post-hoc tests (*p = 0.0136). Histograms compare in MGE cells expressing eGFP and PAK3 mutants, the mean migration speed of the cell body (**C**), the percentage of resting phases (D1) and the frequency (D2, left) and amplitude (D2, right) of nuclear translocations (Parameters defined in Fig. S1E1). Statistical significance is assessed by Kruskal–Wallis tests (all p < 0.0001) and Dunn’s post-hoc tests. **E** Time-lapse sequence shows the GFP signal (top row) during the migration cycle of a control MGE cell expressing the myosin 2B-GFP fusion protein. The GFP intensity is color-coded in the bottom row as indicated on scale. (See also supplementary Movie 2E). **F** Schemes and color-coded pictures illustrate 4 patterns of GFP repeatedly observed in the cell body of migrating MGE cells expressing myosin 2B-GFP: 1, GFP accumulation in the rostral swelling during centrosomal forward migration; 2, GFP concentration at the nuclear rear prior and during nuclear translocation; 3, GFP shift to the nuclear front after nuclear translocation (associated with gliding movement); 4, spreading of small GFP dots in the whole cell body during the resting phase. **G** Histogram representing the frequency of the 4 GFP patterns in MGE cells expressing RFP (black dot), RFP-PAK3-ca (red dot) and RFP-PAK3-kd (blue dot) constructs (See also representative time-lapse sequences in supplementary Fig. S2D, Movies S2D1 and S2D2). Statistical significance of distributions is assessed by chi2 tests. **H** Scheme summarizes the influence of the expression of PAK3 mutants on the morphology, nuclear-centrosomal distance and subcellular localization of myosin 2B in migrating INs.

Owing that the cell body of migrating MGE cells expresses PAK3 as strongly as leading growth cones (Fig. S1C), we analyzed the centrosomal and nuclear movements in MGE cells co-transfected with a fluorescent centrosomal marker (pericentrin-mKO1) and the PAK3 mutants (Fig. 2A). Forward centrosomal movements were significantly shorter in MGE cells co-expressing PAK3-ca than in control MGE cells co-expressing eGFP, and significantly longer in MGE cells co-expressing PAK3-kd (Fig. 2B). Accordingly, the cytoplasmic swelling that accompanies forward centrosomal movements formed less (vs more) frequently in MGE cells expressing PAK3-ca (vs PAK3-kd), as compared to control eGFP MGE cells (not illustrated).

Such opposite changes in nuclear–centrosomal distance did not associate with opposite changes in nuclear migration speed. On the contrary, they both associated with decreased nuclear migration speed (Fig. 2C) due to longer nuclear resting phases and shorter and less frequent nuclear translocations (Fig. 2D1,2; see definition of parameters in Fig. S1D1,2). Therefore, deregulated centrosomal movements in MGE cells expressing either PAK3 mutants associated with nuclear translocation defects, which did not reflect nuclear-centrosomal distance changes (Fig. 2C). Resting phase duration and translocation frequency were not altered in MGE cells expressing PAK3-wt (Fig. S1E). We nevertheless noticed that the saltatory nuclear movements were altered more dramatically in PAK3-ca than in PAK3-kd expressing MGE cells.

The acto-myosin cytoskeleton is known to control nuclear movements and centrosome positioning [37, 42, 45] in migrating neurons. On the other hand, a large set of experimental data has established that PAK kinases regulate the polarized rearrangements of the acto-myosin cytoskeleton involved in cell polarity and cell movement [8, 10, 16, 17, 24]. Because myosin 2B is expressed in tangentially migrating neurons [42, 46], we analyzed the distribution of myosin 2B filaments in MGE cells which have a GFP sequence inserted in the *Myosin 2B* gene (non-muscle myosin 2B-GFP) [39]. MGE cells were moreover electroporated with plasmids encoding PAK3 mutants fused to mRFP (hereafter referred to as RFP-PAK3-ca and RFP-PAK3-kd). The control cells electroporated with mRFP exhibited four stereotyped distribution patterns of GFP(+) myosin2B filaments during migration (Fig. 2E, F, see legend), each correlated with a specific migration speed (Fig. S2A–C). In MGE cells expressing RFP-PAK3-ca, the strong accumulation of GFP(+) myosin 2B filaments at the nuclear rear (pattern 2) was less frequent and compensated by an increased frequency of the faint GFP signal at the nuclear front (pattern 3) (Fig. 2G, red dot and Fig. S2D1). In MGE cells expressing RFP-PAK3-kd, the pattern 3 was rare and compensated by an increased frequency of the patterns 1 and 2 (Fig. 2G, blue dot and Fig. S2D2). Myosin 2B filaments were moreover observed in growth cones before splitting (arrows in Fig. S2D2). In migrating INs, PAK3 kinase activity thus influenced the cyclic pattern of distribution of myosin 2B filaments.

In summary (Fig. 2H), PAK3-ca expressing MGE cells showed shorter forward centrosomal movements and shorter nuclear centrosomal distances than control cells. Myosin 2B filaments assembled less frequently at the nuclear rear, in agreement with the decreased frequency of fast nuclear translocations. On the other hand, PAK3-kd expressing MGE cells showed a decreased nuclear migration speed associated with long forward centrosomal movements in branched leading processes. Myosin 2B filaments assembled in the rostral swelling around the centrosome and around the nucleus, likely preventing nuclear movements. Thus, opposite kinase activity in PAK3-ca and PAK3-kd expressing MGE cells were both associated with decreased nuclear speed.

### Abnormal displacements of MGE cells expressing PAK3 mutants

**Fig. 3:**
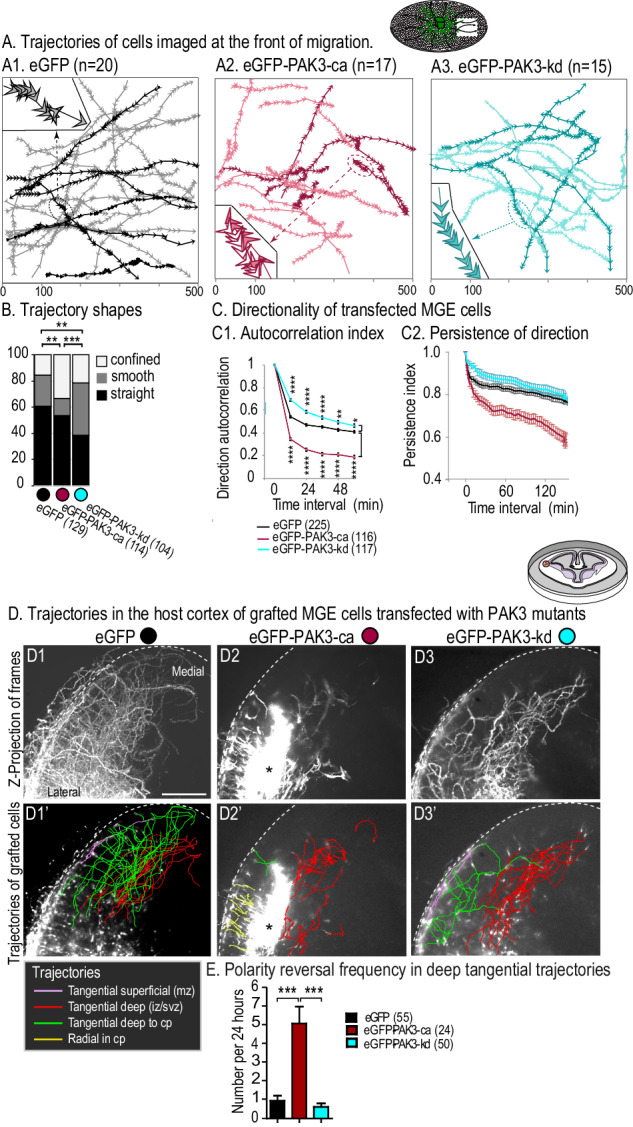
In vitro and ex vivo trajectories of MGE cells electroporated with PAK3 mutants. **A** Frames illustrate the trajectories of MGE cells electroporated with eGFP (A1), eGFP-PAK3-ca (A2) and eGFP-PAK3-kd (A3) constructs migrating away from their explant of origin on wild type dissociated cortical cells. Arrowheads indicate the position and direction of movement of the cell body at each time point. Inserts show an enlarged portion of a trajectory: empty arrowheads represent movements in a direction opposite to the initial direction of the cell. **B** Histogram compares the proportion of straight (black: MGE cells enter and exit the field of view by opposite sides), smoothly curved (grey: cells enter and exit the field of view by adjacent sides) and confined (white: cells remain in the field of view) trajectories. Significance assessed by chi2 tests: p = 0.0077 for eGFP and PAK3-ca comparison, *p* = 0.0054 for EGFP and PAK3-kd comparison, *p* = 0.00015 for PAK3-ca and PAK3-kd comparison. **C** Curves show the autocorrelation index for MGE cells transfected with the different constructs (C1) (cosine of the angular deviation of MGE cell trajectories when the time interval between cell positions increases) and the temporal evolution of the persistence of direction (C2, displacement/real movement ratio). Numbers of analyzed trajectories are indicated. PAK3-ca (resp. PAK3-kd) expressing cells are significantly less (resp. more) directional than control cells, until 80 min. Statistical analysis (two-way ANOVA) of direction autocorrelation: interaction construct × time interval (*F*(10,2730) = 29.32, *p* < 0.0001) and Bonferroni post-hoc tests. **D** Electroporated MGE explants were grafted at the pallium/subpallium boundary of wild-type organotypic forebrain slices (see scheme) and the migration of MGE cells expressing eGFP (D1, D1′), eGFP-PAK3-ca (D2, D2′) or eGFP-PAK3-kd (D3, D′) constructs imaged for 24 h in living cortical slices. D1, D2, D3 are Z-projections of the recorded stacks (see also supplementary Movies 3D1, 3D2, 3D3). D1′, D2′, D3′ show the trajectories of MGE cells tracked manually and colored as indicated in the legend. * indicates the MGE explant in the cortical slice. Scale bar, 300 μm **E** Histogram shows polarity reversal (inversion of nuclear movement direction) frequency in MGE cells expressing eGFP, eGFP-PAK3-ca and eGFP-PAK3-kd (Kruskal-Wallis test, *p* < 0.0001, and Dunn’s post-hoc tests).

We then analyzed the influence of the expression of PAK3 constructs on MGE cell trajectories in co-cultures (Fig. 3A). Most control MGE cells migrated centrifugally away from their origin explant and showed rather straight trajectories (60%, Fig. 3A1, left histogram bar in Fig. 3B) with frequent but very short duration polarity reversals (see enlarged trajectory, Fig. 3A1). MGE cells expressing PAK3-ca showed an increased proportion of confined trajectories (Fig. 3A2, middle histogram bar in Fig. 3B) and exhibited frequent and long duration polarity reversals (see enlarged trajectory, Fig. 3A2). MGE cells expressing PAK3-kd showed an increased proportion of smoothly curved trajectories (Fig. 3A3, right-hand histogram bar in Fig. 3B) and rare polarity reversals (see enlarged trajectory, Fig. 3A3). Accordingly, the angular deviation of trajectories (“direction autocorrelation coefficient” calculated with a published macro [47] in Fig. 3C1, which is close to 1 for straight trajectories) was smaller for cells expressing PAK3-ca and higher for cells expressing PAK3-kd, as compared to control cells. Similarly, the index of direction persistence decreased much faster with time for cells expressing PAK3-ca than for control cells expressing eGFP, and remained above control values for cells expressing PAK3-kd (Fig. 3C2).

We then analyzed the trajectories of MGE cells expressing the PAK3 mutants in a more physiological context, e.g. in organotypic cortical slices that maintain the cortical cytoarchitecture even after more than 1 day in culture. MGE explants electroporated with the different constructs were grafted at the pallium-subpallium boundary in the intermediate zone (see scheme Fig. 3D). Cells migrated away from the laterally positioned graft toward the medial host cortex. Control MGE cells expressing eGFP (Fig. 3D1, D1′) showed tangentially oriented trajectories either in the deep cortical layers (iz/svz, red trajectories) or in the superficial mz (pink trajectories). A large proportion reoriented from the deep tangential pathway to the cp (green trajectories), where they showed radially oriented trajectories. PAK3-ca expressing MGE cells displayed short and erratic trajectories in the presumptive iz/svz (Fig. 3D2, D2′, red) with frequent polarity reversals (inversion of nuclear movement direction, Fig. 3E, red bar), in agreement with their impaired directionality in co-cultures. Remarkably, numerous PAK3-ca expressing cells migrated radially within the cp above the graft (Fig. 3D2′, yellow). By contrast, MGE cells expressing PAK3-kd (Fig. 3D3, D3′) exhibited processive and smoothly curved trajectories in the deep cortical layers (red), in agreement with their smooth trajectories in co-cultures. Very few cells moved to the cp (Fig. 3D3′, green), where they did not re-orient radially (Fig. S3A). MGE cells expressing the PAK3-ca and PAK3-kd mutants both exhibited reduced migration speed in deep cortical layers (red trajectories), despite specific changes in nuclear dynamics (Fig. S3B).

In summary, PAK3-ca expressing cells showed frequent polarity reversals and were much less directional than control cells, in agreement with short nuclear-centrosomal distances and short leading processes. Surprisingly, these cells were able to migrate radially and to stabilize their leading process in the cp of grafted organotypic slices. On the contrary, PAK3-kd expressing MGE cells maintained a stable cell polarity during migration, in agreement with long nuclear-centrosomal distance. According to the production of divergent branches at their front, PAK3-kd expressing MGE cells showed undulating trajectories but hardly invaded the cp in which they did not re-orient radially. This suggests that cp colonization requires polarity changes associated with centrosome re-positioning in the nuclear compartment.

### Altered cortical distribution of MGE cells expressing PAK3 mutants

**Fig. 4:**
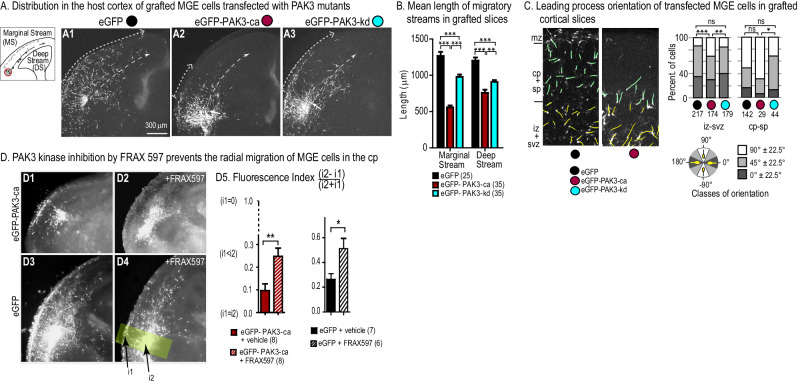
Distribution and leading process orientation of MGE cells expressing PAK3 mutants in grafted cortical slices. **A** MGE explants electroporated with eGFP (A1), eGFP-PAK3-ca (A2) or eGFP-PAK3-kd (A3) constructs were grafted in cortical slices cultured for 36 h and fixed. MGE cells released from the graft organize two tangential streams at the pial surface -marginal stream (light grey) - and along the ventricle -deep stream (black) (scheme on the left). Arrows at the end of the dotted lines indicate the maximum distance reached by electroporated MGE cells in the marginal and deep streams. **B** Histogram of the mean distance between the graft and the most distant electroporated MGE cell, in the marginal (left) and deep (right) streams. Distances between MGE cells expressing eGFP and either PAK3 mutant significantly differ (Two-way ANOVA; interaction migratory stream x electroporated construct, *p* < 0.0001; Bonferroni post-hoc test, *p* < 0.0001) and between MGE cells expressing eGFP-PAK3-ca and eGFP-PAK3-kd (Bonferroni post-hoc test, *p* < 0.001) **C** The leading process orientation (angle with the cortical surface) of grafted MGE cells was classified as radial (90° ± 22.5°, white sector), oblique (45° ± 22.5°, grey sector) or tangential (0° ± 22.5°, black sector). In the intermediate/subventricular zone (iz/svz, leading processes in yellow and left histogram), orientation classes significantly differed between MGE cells expressing eGFP-PAK3-ca and either eGFP (chi2 test, *p* = 0.0009) or eGFP-PAK3-kd (chi2 test, *p* = 0.0038). In the cortical plate/subplate (cp/sp, leading processes in green and right histogram), orientation classes significantly differed between MGE cells expressing eGFP-PAK3-kd and eGFP-PAK3-ca (chi2 test, p = 0.0251). mz marginal zone, vz ventricular zone. **D** MGE explants electroporated with either eGFP-PAK3-ca (D1, D2) or eGFP (D3,D4) were grafted in the iz of cortical slices and cultured in control medium with vehicle (D1, D3) or FRAX597 (D2, D4). The fluorescence index in D5 normalizes and compares the peaks of fluorescence intensity in the grafted area (i2) and in the mz above the graft (i1) (see methods). Index = 0 (resp. = 1) for equal fluorescence intensity in the mz and graft (resp. in the mz and background). FRAX597 treatment significantly diminished the fluorescence intensity in the mz for MGE cells expressing the PAK3-ca construct (Mann–Withney test, *p* = 0.0065) and for MGE cells expressing eGFP (Mann–Withney test, *p* = 0.035).

To further assess the influence of PAK3 activity on cp colonization, we analyzed the distribution of MGE cells expressing PAK3 mutants in a large number of fixed cortical slices (Fig. 4A–C). Cortical slices were grafted with MGE explants electroporated as above, cultured for 36 h and fixed.

In the deep migratory stream (presumptive iz/svz), MGE cells expressing PAK3 mutants distributed over shorter distances than control cells (Fig. 4A1–3, B), in agreement with their reduced migration speed (Fig. S3B1). MGE cells expressing PAK3-ca colonized the presumptive cp above the graft and their tangential dispersion in the marginal stream was minimal as compared with MGE cells expressing eGFP or PAK3-kd (Fig. 4A2, B). Most leading processes of PAK3-ca expressing MGE cells were oriented radially within the cp (90° ± 22.5°, right histogram in Fig. 4C, red dot). By contrast, PAK3-kd expressing MGE cells distributed more largely than PAK3-ca expressing MGE cells in the marginal stream (Fig. 4A3, B). In both deep cortical layers and cp, their leading processes showed mostly oblique and horizontal orientations (45° ± 22.5° or 0 ± 22.5°, histograms in Fig. 4C, blue dots). The distribution of MGE cells expressing eGFP and PAK3 mutants, as well as the orientation of their leading processes, were thus in agreement with the trajectories described in Fig. 3D.

FRAX597, a potent ATP-competitive inhibitor of group I PAKs, which binds to the back cavity of the ATP-binding site of their catalytic domain [41], prevented the radial migration to the cp/mz of PAK3-ca expressing MGE cells grafted in the iz (Fig. 4D1–2, D5 left histogram). Cells appeared blocked within the graft. FRAX597 application on MGE cells expressing eGFP also decreased the proportion of cells able to migrate radially within the cp located above the graft (Fig. 4D3-4, D5 right histogram). In this case however, a few cells were able to migrate tangentially in the iz/svz. Together, these experiments showed that the kinase activity of PAK3 is required for GABAergic INs to enter the cp with a radially oriented trajectory. On the contrary, the inhibition of PAK3 kinase activity promoted tangentially oriented trajectories.

### Abnormal in vivo cortex colonization by MGE cells expressing PAK3 mutants

**Fig. 5:**
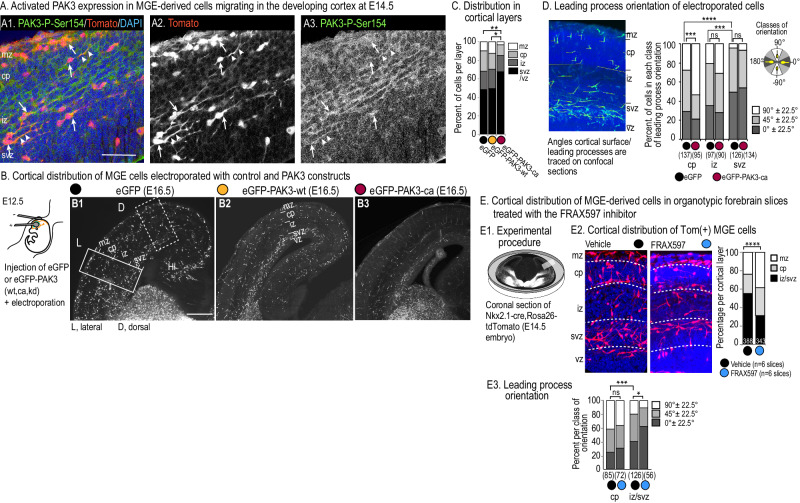
Role of PAK3 kinase activity in the cortical distribution and leading process orientation of MGE-derived interneurons. **A** Immunostaining with anti-activated PAK3(Ser154) antibodies (green) of a frontal cortex section from an E14.5 transgenic embryo expressing tdTomato (red) in MGE-derived cells. E14.5 tdTomato(+) MGE cells exhibit immune-positive lead processes (arrow heads) and/or cell bodies (arrows). Scale bar, 50 μm. **B** Plasmids were injected in the lower part of a lateral ventricle at E12.5, and tweezers positioned to electroporate the medial ganglionic eminence (MGE) of the injected side (See Supplementary methods). Analyses were performed at E16.5 on frontal sections in the cortical region showing the highest density of electroporated cells. B1, B2, B3 are representative pictures of the cortical distribution of MGE cells electroporated with eGFP (B1), eGFP-PAK3-wt (B2) or eGFP-PAK3-ca (B3) constructs. Scale bar, 300 μm. **C** The cortical distribution of PAK3-ca expressing MGE cells significantly differed from the distributions of MGE cells expressing eGFP (chi2 test, *p* = 0.0046) and PAK3-wt (chi2 test, *p* = 0.0148). **D** Picture shows a DAPI counterstained cortical section containing electroporated cells. Section was imaged by confocal microscopy and leading process orientation measured on the Z-projection of a stack acquired every 2 µm over 30 µm. Histograms compare the distribution in the 3 classes of orientation of EGFP and PAK3-ca expressing GE cells located in different cortical layers. Radially oriented EGFP(+) GE cells were significantly more numerous in the cp and iz than in the svz (chi2 tests, *** *p* = 0.0007). Orientations of EGFP(+) and PAK3-ca(+) cells significantly differed in the cp (chi2 test, *p* = 0.0003). **E** Ex vivo analysis of the cortical distribution of MGE-derived cells in the presence of the pharmacological inhibitor FRAX597. E1. Forebrain slices from transgenic embryos were cultured in Millicell chambers in the presence of either FRAX597 or vehicle. E2. Pictures are Z-projections of confocal stacks of a cortical sector (E1). DAPI counterstaining revealed cortical layers. The number of Tomato(+) MGE-derived cells decreased in FRAX597-treated slices and their layer distribution changed (chi2 test, *p* < 0.0001). E3. Histograms show that tangentially oriented Tomato(+) MGE-derived cells were significantly more numerous in the iz/svz of FRAX597-treated than control slices (chi2 test, *p* = 0.0215). Leading process orientations in cortical layers of control slices were compared with chi2 tests (***p = 0.0007). mz marginal zone, cp cortical plate, sp subplate, iz intermediate zone, svz subventricular zone, vz ventricular zone.

A previous study by Cobos et al. [23] established that *Pak3* is already expressed in the mouse forebrain at E11.5 and that MGE-derived cortical INs up regulate *Pak3* expression as they enter the developing cortex. We showed that *Pak3* was strongly expressed in the proliferative areas of the lateral (LGE) and caudal (CGE) GEs, and in the mantle zone of the GEs at E12.5–E14.5, when young GABAergic MGE cells colonize the cortical anlage (Fig. S4A1-C2). Staining of E13.5–E14.5 forebrain sections with PAK3 antibodies confirmed the presence of immunopositive PAK3+ cells with tangentially oriented processes in the tangential migratory pathways of cINs (mz, iz) (Fig. S4D–E2). PAK3+ cortical progenitors moreover distributed along the ventricle (Fig. S4D–E2). An antibody that recognizes endogenous activated PAK3 (anti-PAK3-PhosphoSer154) labeled as previously reported [25], a dense network of tangentially oriented fibers in the cortical iz, cells and processes in the mz (Fig. S4F1–3) and cortical progenitors along the ventricle (Fig. S4G1–3). Confocal analyses revealed immunostaining in the leading process and perinuclear compartment of Tomato expressing MGE-derived cells (Fig. 5A1–3).

We then performed in utero electroporation experiments to examine in vivo the influence of the kinase activity of PAK3 on the cortical distribution of MGE cells that colonize the cortex at E13.5–E14.5. Plasmids were electroporated unilaterally in the MGE of mouse embryos at E12.5 [42] and the cortical distribution of transfected MGE cells was analyzed at E16.5 (Fig. 5B). To our surprise, among the 30 surviving embryos electroporated with the Pak3-kd construct only 2 embryos showed electroporated MGE cells in the dorsal cortex (Table 1, Fig. S5A). Remarkably, neither the death rate of electroporated embryos (Table 1), nor the cell death of PAK3-kd expressing MGE cells (Fig. S5B1–3) were increased. Notably, PAK3-ca expressing MGE cells whose death rate was increased in vitro (Fig. S5B2) remained able to reach the dorsal cortex of electroporated embryos. Therefore, cell death unlikely explained the absence of PAK3-kd expressing MGE cells in the dorsal cortex of electroporated embryos. To further explore the capacity of PAK3-kd expressing MGE cells to migrate from the basal forebrain to the cortex, we grafted PAK3-kd expressing MGE cells in the MGE of organotypic slices (Fig. S5C). Grafted cells did not reached the dorsal cortex of slices, in total agreement with the absence of PAK3-kd expressing MGE cells in the dorsal cortex of electroporated embryos. In grafted slices, PAK3-kd expressing MGE cells organized dense tangential migratory streams in the basal telencephalon (Fig. S5C2), and a minimal number of transfected cells entered the dorsal cortex (Fig. S5C3). We thus propose that PAK3-kd expressing MGE cells in the electroporated embryos could remain in the migratory pathways of the basal forebrain and hardly colonize the dorsal cortex.

**Table 1:** Numbers of embryos electroporated in the ganglionic eminences (GEs) at E12.5 with the different constructs.

Plasmids	Nber of electroporated embryos	Nber of living embryos	Nber of surviving embryos with cortical streams of electroporated GE cells	Mean Nber of cells/embryo (lateral + dorsal sectors)
pCAG-eGFP	32 (3 litters)	27 (84%)	12 (44% of surviving)	195 ± 45
pCAG-eGFP-PAK3-wt	34 (4 litters)	17 (50%)	6 (35% of surviving)	103 ± 22
pCAG-eGFP-PAK3-ca	51 (5 litters)	28 (55%)	7 (25% of surviving)	58 ± 18
pCAG-eGFP-PAK3-kd	39 (5 litters)	30 (77%)	2 (7% of surviving)	

The table shows the numbers and percentages of embryos that survived until E16.5 and in which electroporated GE cells reached the cortex.

Whereas the cortical distribution at E16.5 of MGE cells electroporated with the control eGFP and PAK3-wt constructs resembled each other (large migratory stream in svz, thinner migratory stream in mz, and similar distribution in-between in cp and iz, Fig. 5B1, 2, C), most MGE cells electroporated with the PAK3-ca construct remained in the deep cortical layers (Fig. 5C). PAK3-ca expressing MGE cells that reached the cp showed more frequently radially oriented leading processes than eGFP- expressing MGE cells (Fig. 5D).

To further assess the influence of PAK3 kinase inhibition on the distribution of MGE cells in the cortical layers, we cultured forebrain slices from E14.5 transgenic embryos with Tomato(+) MGE-derived cells, in the presence of FRAX597 (Fig. 5E). The proportion of Tomato(+) MGE-derived cells very significantly decreased in the deep layers (iz/svz) of FRAX597 treated slices (Fig. 5E2) and the leading process of these cells was more frequently tangentially oriented than in control slices (Fig. 5E3), suggesting that inhibiting PAK3 kinase activity promoted IN tangential movements in these layers.

Altogether, these results showed that PAK3 kinase activity in INs migrating in the deep cortical layers impairs tangential progression and promotes trajectory reorientation.

**Fig. 6:**
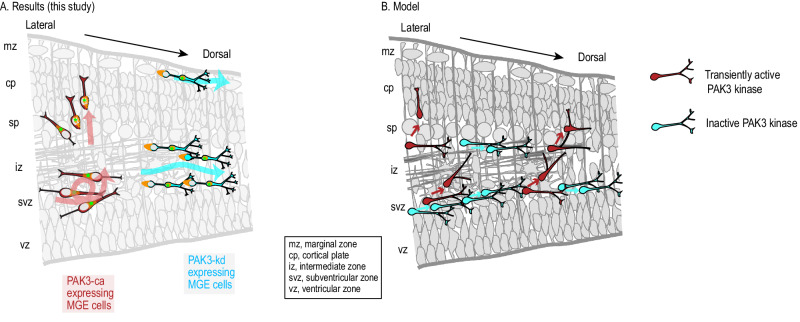
Summary. The scheme on the left (**A**) is a graphical summary of the present study: the expression of the constitutively active PAK3 kinase is responsible for IN morphological changes schematized by red cells and trajectory re-orientations in the upper and lower cortical layers figured by the thick red lines; the expression of a non-activable PAK3 kinase is responsible for opposite morphological changes schematized by blue cells and for the formation of large tangential migratory streams of INs figured by the thick blue lines. Centrosome in green and myosin 2B filaments in orange. The scheme on the right (**B**) is an interpretative model of our results. The transient activation of PAK3 kinase in a subset of tangentially migrating INs disrupts the cell polarity and leading process stability in these cells, thereby preparing them to respond to extrinsic guidance cues that promote their re-orientation away from the tangential streams towards the cortical plate. By contrast, INs with inactive PAK3 should maintain a tangential progression.

In conclusion, our ex vivo and in vivo studies show that INs expressing a kinase activated PAK3 variant operate a tangential to radial switch of migration required for cp colonization (summary in Fig. 6A). These INs present unstable cell polarity due to decreased centrosomal-nuclear distance and decreased leading process length and stability. Remarkably, the insertion of PAK3-ca expressing INs within the cp stabilizes radially oriented processes. By contrast, INs expressing the non-activable PAK3 variant are unable to re-orient their trajectory radially, to insert in the radially oriented cp. They maintain a tangential forward migration (Fig. 6A). The transient activity of PAK3 kinase could allow migrating INs to re-orient and escape their tangential migratory streams (see Model in Fig. 6B).

## Discussion

The present study aimed at characterizing the role of the PAK3 kinase in the cortical migration of GABAergic INs. By combining cellular and subcellular studies of migrating INs in co-culture models, we demonstrated an important role for the PAK3 kinase activity in the control of the leading process stability and of the cell polarity of migrating INs. By analyzing the distribution, orientation and trajectories of transfected INs in organotypic cortical slices and electroporated embryos, we uncovered a decisive role of these two cellular properties, e.g. leading process stability and cell polarity, to control the tangential progression versus the tangential to radial re-orientation of cortical INs in the developing cortex.

### PAK3 kinase activity controls leading process morphology and leading process orientation in INs

INs expressing PAK3 variants differed from control INs and from each other by their leading process morphology, in line with their growth cone dynamics. Previous studies showed that the reorientation of cortical INs from tangential pathways to the cp was accompanied by drastic morphological changes of leading processes that extended novel protrusions towards the cp while they retracted tangentially oriented processes [35, 36]. INs expressing PAK3-kd extended branched leading processes responsible for moderate angular deviations of trajectories and showed smoothly curved trajectories within the deep tangential pathway of cortical slices. By contrast, INs expressing PAK3-ca frequently changed their cell polarity and unstable cell polarity favored major trajectory reorientations such as the tangential to radial switch. Such directional changes could be triggered by guidance cues, either attractive or repulsive, distributed within the developing cortex [48, 49]. In this hypothesis, INs expressing PAK3-kd would remain blind to those signals, while INs expressing PAK3-ca would strongly respond to them. After entering the cp, INs expressing PAK3-ca maintained a radial orientation along the cp cells, suggesting that extrinsic cp signals, comprising adhesive interactions with cp cells, stabilize their insertion within the cp [50].

RAC1/3 and CDC42 are the major activators of PAK kinases. Migrating cortical INs express both *Rac1* and *Rac3* that exert partly redundant functions [51]. Several guidance cues able to activate Rac are moreover known to influence IN trajectories within the cortex [49, 51–53]. Some morphological abnormalities observed in Rac1/3 deleted INs recall those of INs expressing the PAK3-kd mutant, in particular the increased number of neurites and a very remarkable splitting of the leading process [54]. Despite smaller growth cones, the motility of PAK3-kd expressing INs in the tangential cortical pathways remained unchanged, as previously described in *Rac* depleted INs [54, 55]. By contrast, PAK3-kd expressing INs hardly crossed the pallium-subpallium boundary in electroporated embryonic brains, a default that recalls the impaired migration of INs from the basal to the dorsal forebrain in *Rac1/3* double KOs [54]. Whether RAC can promote the radial re-orientation of INs by activating PAK3 is an open question. RAC1 has been shown to regulate the morphology and dynamics of the leading process of radially migrating cells [56, 57]. We previously showed that N-cadherin mediates interactions between migrating INs and radially oriented cells in the cp [42].The role of PAK3 in regulating these interactions remains to be explored. CDC42 has already been identified as a PAK3 activator in cortical neurons [19]. CDC42 cooperates with RAC at the leading edge of moving cells to regulate cell adhesion and actin dynamic [13, 14]. In addition, CDC42 has been shown to promote the extension and to stabilize protrusions in the developing cortex [58]. Both GTPases could cooperate or successively activate PAK3 to control and stabilize the radial orientation of IN processes in the cp.

### PAK3 kinase activity regulates cell polarity by controlling the nucleus-centrosome distance

PAKs, in particular PAK1, control cell polarity in neurons and non-neural cells downstream of RAC, CDC42, and PIX –GIT complexes [10]. In migrating cells, the polarity is stabilized by the positioning of the centrosome in front of the nucleus at the basis of the leading process. Our results identify the nucleus-centrosome distance as a master regulator of cell polarity and sustained directionality in migrating cortical INs.

The precise role of PAK3 in controlling the centrosome position in migrating INs remains unclear. PAK can be recruited and activated at the centrosome for regulating signaling pathways involved in cell division [59] (See Fig. S4G1–3). CDC42, which has been shown to accumulate in the centrosomal region of migrating neurons [56], is largely involved in the activation of a variety of signaling pathways regulating cell polarity and directed migration [12–14]. However, the capacity of CDC42 to activate PAKs near the centrosome is not known. Cerebellar granule cells invalidated for *Cdc42* or overexpressing a dominant negative CDC42 show polarity defaults and impaired junctions with Bergman glia associated with phosphorylation changes in PAK proteins. The expression level of the protein complex that regulates centrosomal movements in these neurons remained unchanged [60, 61]. POSH, an activator of RAC1, has also been localized in the rostral swelling of radially migrating cortical neurons, where the centrosome positions before nuclear translocation [62].

PAK proteins are important regulators of the acto-myosin and microtubule cytoskeleton. They have long been recognized to control cell motility, cell contractility and tension in tissues by regulating the dynamics of the actomyosin cytoskeleton [8, 10, 16, 17]. Myosin 2B is a non-muscle myosin II isoform that defines the non-protrusive parts of migrating cells -nuclear compartment and trailing edge- and regulates nuclear dynamics [63]. Myosin 2B seems functionally predominant in tangentially migrating neurons [46]. By monitoring a non-muscle myosin 2B-GFP fusion protein [39] in PAK3-ca and PAK3-kd expressing INs, we indeed showed that PAK3 kinase activity significantly altered the subcellular distribution of the non-muscle myosin 2B filaments in migrating cortical INs and regulated the acto-myosin dependent contractility of the cell body. Accordingly to studies performed in contractile non-muscle cells [64], no direct effect of PAK3 kinase on regulatory kinases of the myosin 2B was shown (data not shown). This suggests that complex regulatory mechanisms targeting other PAK targets (actin filaments, adhesion sites,…) could mediate the effect of PAK3 kinase activity on the cyclic assembly and distribution of myosin 2B filaments in migrating INs. In motile cells, PAKs could control microtubule dynamics by blocking the microtubule depolymerizing activity of stathmin through phosphorylation [20], thereby regulating the length and stability of microtubule bundles linking the centrosomal swelling to the nuclear compartment and to the leading process [40, 45].

### PAK3 kinase activity and PAK3 expression level both regulate the migration of cortical INs

Cobos et al. [23] previously reported that acute *Pak3* overexpression does not affect the neuritic growth in wild type INs, whereas constitutive *Pak3* overexpression in *Dlx1-/-Dlx2-/-* INs associates with longer neurites and increased dendritic complexity. In this model, the tangential migration was moreover abrogated and defects were rescued by Pak3 siRNAs. INs with either *Dlx1* or *Foxg1* deletion, that both promote *Pak3* overexpression, presented similar increase in neurite growth and dendritic complexity [26, 27]. Whether *Pak3* overexpression correlates with increased kinase activity has not been tested in these animal models.

In the present study, we show that INs expressing an active PAK3 mutant stopped their tangential migration as reported in *Dlx1/2 −/−* INs. However, leading processes were shortened, a defect opposite to that observed in *Dlx1/2 −/−* INs. Contradictory results have also been reported in cell lines overexpressing activated PAK1: numerous protrusions and increased migration were observed in some cells [16], stable focal adhesions and impaired migration in other cell types [17]. As suggested in cell lines, the influence of PAK activity on the neuritic growth could depend on the physiological state of the INs, either migrating (this study) or differentiating [23, 26, 27].

It remains that PAK3 functions are moreover controlled by expression level and transcriptional regulation. In vivo, PAK3 expression in cortical INs is indeed subject to a complex regulation by transcription factors (*Dlx, Ash1, Sp8/9*) [26, 27, 65]. MGE-derived INs up-regulate *Pak3* as they enter the cortex [23]. IN migration is a complex and multistep process that could be regulated by both PAK3 expression and PAK3 kinase activity. In addition, Combeau et al. [66] showed that PAK3 preferentially dimerizes with PAK1, which is expressed at higher levels than PAK3 in the developing brain [10, 66]. Interestingly, *PAK1* mutations also associate with intellectual disability, severe epilepsy, autism spectrum disorder and macrocephaly [67–69]. Cortical GABAergic neurons likely express PAK1 in addition to PAK3 [24]. PAK3 overexpression in cortical INs could thus significantly interfere with PAK1 activation. Reciprocally, a putative “buffering” effect of PAK1 on PAK3 overexpression remains to be investigated. Analyses of *Pak3* KO and *Pak1/Pak3* double KO mice support important interactions between both isoforms [22, 70]. Compensatory effects by regulatory complexes could better attenuate the effect of PAK3 depletion than that of point mutations.

## Conclusion

In the present study, we have identified PAK3 as a kinase whose activity in cortical INs interrupts the tangential migration and promotes the radial re-orientation and migration within the cp. All together, our results suggest that cp colonization by INs relies on the capacity for PAK3 kinase to switch from an inactive to an active state. PAK3 activity disrupts the polarized morphology that allows INs to present a persistent tangential migration by preventing the formation and stabilization of branches in which the centrosome migrates forward, and by impairing centrosome stabilization at a distance from the nucleus. PAK3 is strongly activated in the mz and iz, the two routes in which growing axons and migrating cells move tangentially and multiple signals could trigger PAK3 activation in these tangential cortical pathways. PAK3 mutations targeting the kinase domain of the protein, most frequently found in patients with intellectual disability and cortical malformations, should therefore severely impair the migration of cortical INs in infants and the formation of normal cortical circuits.

## Supplementary information

Supplementary information (docx file)

Movie S1A

Movie 2E

Movie S2D1

Movie S2D2

Movie 3D1

Movie 3D2

Movie 3D3

## Data Availability

The data that support the findings of this study are available from the corresponding author upon reasonable request. Data are stored at INSERM U1270.

## References

[1] Allen KM, Gleeson JG, Bagrodia S, Partington MW, MacMillan JC, Cerione RA, et al. PAK3 mutation in nonsyndromic X-linked mental retardation. Nat Genet. 1998;20:25–30. 9731525 10.1038/1675

[2] Rejeb I, Saillour Y, Castelnau L, Julien C, Bienvenu T, Taga P, et al. A novel splice mutation in PAK3 gene underlying mental retardation with neuropsychiatric features. Eur J Hum Genet. 2008;16:1358–63. 18523455 10.1038/ejhg.2008.103

[3] Magini P, Pippucci T, Tsai I-C, Coppola S, Stellacci E, Bartoletti-Stella A, et al. A mutation in PAK3 with a dual molecular effect deregulates the RAS/MAPK pathway and drives an X-linked syndromic phenotype. Hum Mol Genet. 2014;23:3607–17. 24556213 10.1093/hmg/ddu070

[4] Horvath GA, Tarailo-Graovac M, Bartel T, Race S, Van Allen MI, Blydt-Hansen I, et al. Improvement of self-injury with dopamine and serotonin replacement therapy in a patient with a hemizygous PAK3 mutation: a new therapeutic strategy for neuropsychiatric features of an intellectual disability syndrome. J Child Neurol. 2018;33:106–13. 29246092 10.1177/0883073817740443

[5] Iida A, Takano K, Takeshita E, Abe-Hatano C, Hirabayashi S, Inaba Y, et al. A novel PAK3 pathogenic variant identified in two siblings from a Japanese family with X-linked intellectual disability: case report and review of the literature. Cold Spring Harb Mol Case Stud. 2019;5:a003988. 31444167 10.1101/mcs.a003988 PMC6913141

[6] Qian Y, Wu B, Lu Y, Zhou W, Wang S, Wang H. Novel PAK3 gene missense variant associated with two Chinese siblings with intellectual disability: a case report. BMC Med Genet. 2020;21:31–8. 32050918 10.1186/s12881-020-0957-x PMC7017536

[7] Duarte K, Heide S, Poëa-Guyon S, Rousseau V, Depienne C, Rastetter A, et al. PAK3 mutations responsible for severe intellectual disability and callosal agenesis inhibit cell migration. Neurobiol Dis. 2020;136:104709. 31843706 10.1016/j.nbd.2019.104709

[8] Bokoch GM. Biology of the p21-activated kinases. Annu Rev Biochem. 2003;72:743–81. 12676796 10.1146/annurev.biochem.72.121801.161742

[9] Arias-Romero LE, Chernoff J. A tale of two Paks. Biol Cell. 2008;100:97–108. 18199048 10.1042/BC20070109

[10] Kreis P, Barnier J-V. PAK signalling in neuronal physiology. Cell Signal. 2009;21:384–93. 19036346 10.1016/j.cellsig.2008.11.001

[11] Zhang K, Wang Y, Fan T, Zeng C, Sun ZS. The p21-activated kinases in neural cytoskeletal remodeling and related neurological disorders. Protein Cell. 2022;13:6–25. 33306168 10.1007/s13238-020-00812-9 PMC8776968

[12] Azzarelli R, Kerloch T, Pacary E. Regulation of cerebral cortex development by Rho GTPases: insights from in vivo studies. Front Cell Neurosci. 2014;8:445. 25610373 10.3389/fncel.2014.00445 PMC4285737

[13] Etienne-Manneville S. Polarity proteins in migration and invasion. Oncogene. 2008;27:6970–80. 19029938 10.1038/onc.2008.347

[14] Govek E-E, Hatten ME, Van Aelst L. The role of Rho GTPase proteins in CNS neuronal migration. Dev Neurobiol. 2011;71:528–53. 21557504 10.1002/dneu.20850 PMC3188326

[15] Lawson CD, Ridley AJ. Rho GTPase signaling complexes in cell migration and invasion. J Cell Biol. 2018;217(2):447–57. 29233866 10.1083/jcb.201612069 PMC5800797

[16] Sells MA, Boyd JT, Chernoff J. p21-activated kinase 1 (Pak1) regulates cell motility in mammalian fibroblasts. J Cell Biol. 1999;145:837–49. 10330410 10.1083/jcb.145.4.837 PMC2133181

[17] Kiosses WB, Daniels RH, Otey C, Bokoch GM, Schwartz MA. A role for p21-activated kinase in endothelial cell migration. J Cell Biol. 1999;147:831–44. 10562284 10.1083/jcb.147.4.831 PMC2156168

[18] Jacobs T, Causeret F, Nishimura YV, Terao M, Norman A, Hoshino M, et al. Localized activation of p21-activated kinase controls neuronal polarity and morphology. J Neurosci. 2007;27:8604–15. 17687038 10.1523/JNEUROSCI.0765-07.2007 PMC2080820

[19] Kreis P, Thevenot E, Rousseau V, Boda B, Muller D, Barnier J-V. The p21-activated kinase 3 implicated in mental retardation regulates spine morphogenesis through a Cdc42-dependent pathway. J Biol Chem. 2007;282:21497–506. 17537723 10.1074/jbc.M703298200

[20] Kwon Y, Jeon YW, Kwon M, Cho Y, Park D, Shin JE. βPix-d promotes tubulin acetylation and neurite outgrowth through a PAK/Stathmin1 signaling pathway. PLoS ONE. 2020;15:e0230814. 32251425 10.1371/journal.pone.0230814 PMC7135283

[21] Hayashi K, Ohshima T, Mikoshiba K. Pak1 is involved in dendrite initiation as a downstream effector of Rac1 in cortical neurons. Mol Cell Neurosci. 2002;20:579–94. 12213441 10.1006/mcne.2002.1144

[22] Meng J, Meng Y, Hanna A, Janus C, Jia Z. Abnormal long-lasting synaptic plasticity and cognition in mice lacking the mental retardation gene Pak3. J Neurosci. 2005;25:6641–50. 16014725 10.1523/JNEUROSCI.0028-05.2005 PMC6725420

[23] Cobos I, Borello U, Rubenstein JLR. Dlx transcription factors promote migration through repression of axon and dendrite growth. Neuron. 2007;54:873–88. 17582329 10.1016/j.neuron.2007.05.024 PMC4921237

[24] Causeret F, Terao M, Jacobs T, Nishimura YV, Yanagawa Y, Obata K, et al. The p21-activated kinase is required for neuronal migration in the cerebral cortex. Cereb Cortex. 2009;19:861–75. 18701438 10.1093/cercor/bhn133 PMC2651475

[25] Demyanenko GP, Halberstadt AI, Rao RS, Maness PF. CHL1 cooperates with PAK1-3 to regulate morphological differentiation of embryonic cortical neurons. Neuroscience. 2010;165:107–15. 19819308 10.1016/j.neuroscience.2009.09.077 PMC2791357

[26] Dai X, Iwasaki H, Watanabe M, Okabe S. Dlx1 transcription factor regulates dendritic growth and postsynaptic differentiation through inhibition of neuropilin-2 and PAK3 expression. Eur J Neurosci. 2014;39:531–47. 24236816 10.1111/ejn.12413

[27] Shen W, Ba R, Su Y, Ni Y, Chen D, Xie W, et al. Foxg1 regulates the postnatal development of cortical interneurons. Cereb Cortex. 2019;29:1547–60. 29912324 10.1093/cercor/bhy051 PMC6676970

[28] Marín O. Interneuron dysfunction in psychiatric disorders. Nat Rev Neurosci. 2012;13:107–20. 22251963 10.1038/nrn3155

[29] Wonders CP, Anderson SA. The origin and specification of cortical interneurons. Nat Rev Neurosci. 2006;7:687–96. 16883309 10.1038/nrn1954

[30] Marín O, Rubenstein JLR. Cell migration in the forebrain. Annu Rev Neurosci. 2003;26:441–83. 12626695 10.1146/annurev.neuro.26.041002.131058

[31] Yozu M, Tabata H, Nakajima K. The caudal migratory stream: a novel migratory stream of interneurons derived from the caudal ganglionic eminence in the developing mouse forebrain. J Neurosci. 2005;25:7268–77. 16079409 10.1523/JNEUROSCI.2072-05.2005 PMC6725225

[32] Marín O, Valiente M, Ge X, Tsai L-H. Guiding neuronal cell migrations. Cold Spring Harb Perspect Biol. 2010;2:a001834. 20182622 10.1101/cshperspect.a001834 PMC2828271

[33] Tanaka D, Nakaya Y, Yanagawa Y, Obata K, Murakami F. Multimodal tangential migration of neocortical GABAergic neurons independent of GPI-anchored proteins. Development. 2003;130:5803–13. 14534141 10.1242/dev.00825

[34] Faux C, Rakic S, Andrews W, Britto JM. Neurons on the move: migration and lamination of cortical interneurons. Neurosignals. 2012;20:168–89. 22572780 10.1159/000334489

[35] Martini FJ, Valiente M, López-Bendito G, Szabo G, Moya F, Valdeolmillos M, et al. Biased selection of leading process branches mediates chemotaxis during tangential neuronal migration. Development. 2009;136:41–50. 19060332 10.1242/dev.025502

[36] Lysko DE, Putt M, Golden JA. SDF1 regulates leading process branching and speed of migrating interneurons. J Neurosci. 2011;31:1739–45. 21289183 10.1523/JNEUROSCI.3118-10.2011 PMC3038431

[37] Peyre E, Silva CG, Nguyen L. Crosstalk between intracellular and extracellular signals regulating interneuron production, migration and integration into the cortex. Front Cell Neurosci. 2015;9:129. 25926769 10.3389/fncel.2015.00129 PMC4396449

[38] Long JZ, Lackan CS, Hadjantonakis A-K. Genetic and spectrally distinct in vivo imaging: embryonic stem cells and mice with widespread expression of a monomeric red fluorescent protein. BMC Biotechnol. 2005;5:20. 15996270 10.1186/1472-6750-5-20 PMC1192791

[39] Bao J, Ma X, Liu C, Adelstein RS. Replacement of nonmuscle myosin II-B with II-A rescues brain but not cardiac defects in mice. J Biol Chem. 2007;282:22102–11. 17519229 10.1074/jbc.M702731200

[40] Baudoin J-P, Viou L, Launay P-S, Luccardini C, Espeso Gil S, Kiyasova V, et al. Tangentially migrating neurons assemble a primary cilium that promotes their reorientation to the cortical plate. Neuron. 2012;76:1108–22. 23259947 10.1016/j.neuron.2012.10.027

[41] Licciulli S, Maksimoska J, Zhou C, Troutman S, Kota S, Liu Q, et al. FRAX597, a small molecule inhibitor of the p21-activated kinases, inhibits tumorigenesis of neurofibromatosis type 2 (NF2)-associated Schwannomas. J Biol Chem. 2013;288:29105–14. 23960073 10.1074/jbc.M113.510933 PMC3790009

[42] Luccardini C, Hennekinne L, Viou L, Yanagida M, Murakami F, Kessaris N, et al. N-Cadherin sustains motility and polarity of future cortical interneurons during tangential migration. J Neurosci. 2013;33:18149–60. 24227724 10.1523/JNEUROSCI.0593-13.2013 PMC3858641

[43] Rousseau V, Goupille O, Morin N, Barnier JV. A new constitutively active brain PAK3 isoform displays modified specificities toward Rac and Cdc42 GTPases. J Biol Chem. 2003;278:3912–20. 12464619 10.1074/jbc.M207251200

[44] Bellion A, Baudoin J-P, Alvarez C, Bornens M, Métin C. Nucleokinesis in tangentially migrating neurons comprises two alternating phases: forward migration of the Golgi/centrosome associated with centrosome splitting and myosin contraction at the rear. J Neurosci. 2005;25:5691–9. 15958735 10.1523/JNEUROSCI.1030-05.2005 PMC6724882

[45] Solecki DJ, Trivedi N, Govek E-E, Kerekes RA, Gleason SS, Hatten ME. Myosin II motors and F-actin dynamics drive the coordinated movement of the centrosome and soma during CNS glial-guided neuronal migration. Neuron. 2009;63:63–80. 19607793 10.1016/j.neuron.2009.05.028 PMC2737100

[46] Ma X, Kawamoto S, Hara Y, Adelstein RS. A point mutation in the motor domain of nonmuscle myosin II-B impairs migration of distinct groups of neurons. Mol Biol Cell. 2004;15:2568–79. 15034141 10.1091/mbc.E03-11-0836 PMC420083

[47] Gorelik R, Gautreau A. Quantitative and unbiased analysis of directional persistence in cell migration. Nat Protoc. 2014;9:1931–43. 25033209 10.1038/nprot.2014.131

[48] Tiveron M-C, Rossel M, Moepps B, Zhang YL, Seidenfaden R, Favor J, et al. Molecular interaction between projection neuron precursors and invading interneurons via stromal-derived factor 1 (CXCL12)/CXCR4 signaling in the cortical subventricular zone/intermediate zone. J Neurosci. 2006;26:13273–8. 17182777 10.1523/JNEUROSCI.4162-06.2006 PMC6674999

[49] Zimmer G, Rudolph J, Landmann J, Gerstmann K, Steinecke A, Gampe C, et al. Bidirectional ephrinB3/EphA4 signaling mediates the segregation of medial ganglionic eminence- and preoptic area-derived interneurons in the deep and superficial migratory stream. J Neurosci. 2011;31:18364–80. 22171039 10.1523/JNEUROSCI.4690-11.2011 PMC6623906

[50] Elias LAB, Turmaine M, Parnavelas JG, Kriegstein AR. Connexin 43 mediates the tangential to radial migratory switch in ventrally derived cortical interneurons. J Neurosci. 2010;30:7072–7. 20484649 10.1523/JNEUROSCI.5728-09.2010 PMC2883285

[51] de Curtis I. Roles of Rac1 and Rac3 GTPases during the development of cortical and hippocampal GABAergic interneurons. Front Cell Neurosci. 2014;8:307. 25309333 10.3389/fncel.2014.00307 PMC4174739

[52] DeGeer J, Boudeau J, Schmidt S, Bedford F, Lamarche-Vane N, Debant A. Tyrosine phosphorylation of the Rho guanine nucleotide exchange factor Trio regulates netrin-1/DCC-mediated cortical axon outgrowth. Mol Cell Biol. 2013;33:739–51. 23230270 10.1128/MCB.01264-12 PMC3571336

[53] Stanco A, Szekeres C, Patel N, Rao S, Campbell K, Kreidberg JA, et al. Netrin-1-alpha3beta1 integrin interactions regulate the migration of interneurons through the cortical marginal zone. Proc Natl Acad Sci USA. 2009;106:7595–7600. 19383784 10.1073/pnas.0811343106 PMC2678634

[54] Tivodar S, Kalemaki K, Kounoupa Z, Vidaki M, Theodorakis K, Denaxa M, et al. Rac-GTPases regulate microtubule stability and axon growth of cortical gabaergic interneurons. Cereb Cortex. 2015;25:2370–82. 24626607 10.1093/cercor/bhu037 PMC4537417

[55] Vidaki M, Tivodar S, Doulgeraki K, Tybulewicz V, Kessaris N, Pachnis V, et al. Rac1-dependent cell cycle exit of MGE precursors and GABAergic interneuron migration to the cortex. Cereb Cortex. 2012;22:680–92. 21690261 10.1093/cercor/bhr145 PMC3589917

[56] Konno D, Yoshimura S, Hori K, Maruoka H, Sobue K. Involvement of the phosphatidylinositol 3-kinase/Rac1 and Cdc42 pathways in radial migration of cortical neurons. J Biol Chem. 2005;280:5082–8. 15557338 10.1074/jbc.M408251200

[57] Kawauchi T, Chihama K, Nabeshima Y-I, Hoshino M. The in vivo roles of STEF/Tiam1, Rac1 and JNK in cortical neuronal migration. EMBO J. 2003;22:4190–201. 12912917 10.1093/emboj/cdg413 PMC175802

[58] Cappello S, Attardo A, Wu X, Iwasato T, Itohara S, Wilsch-Bräuninger M, et al. The Rho-GTPase cdc42 regulates neural progenitor fate at the apical surface. Nat Neurosci. 2006;9:1099–107. 16892058 10.1038/nn1744

[59] Zhao Z-S, Lim JP, Ng Y-W, Lim L, Manser E. The GIT-associated kinase PAK targets to the centrosome and regulates aurora-A. Mol Cell. 2005;20:237–49. 16246726 10.1016/j.molcel.2005.08.035

[60] Govek E-E, Wu Z, Acehan D, Molina H, Rivera K, Zhu X, et al. Cdc42 regulates neuronal polarity during cerebellar axon formation and glial-guided migration. iScience. 2018;1:35–48. 29888765 10.1016/j.isci.2018.01.004 PMC5993048

[61] Solecki DJ, Model L, Gaetz J, Kapoor TM, Hatten ME. Par6α signaling controls glial-guided neuronal migration. Nat Neurosci. 2004;7:1195–203. 15475953 10.1038/nn1332

[62] Yang T, Sun Y, Zhang F, Zhu Y, Shi L, Li H, et al. POSH localizes activated Rac1 to control the formation of cytoplasmic dilation of the leading process and neuronal migration. Cell Rep. 2012;2:640–51. 22959435 10.1016/j.celrep.2012.08.007

[63] Vicente-Manzanares M, Ma X, Adelstein RS, Horwitz AR. Non-muscle myosin II takes centre stage in cell adhesion and migration. Nat Rev Mol Cell Biol. 2009;10:778–90. 19851336 10.1038/nrm2786 PMC2834236

[64] Zhang W, Huang Y, Gunst SJ. p21-activated kinase (Pak) regulates airway smooth muscle contraction by regulating paxillin complexes that mediate actin polymerization. J Physiol. 2016;594:4879–4900. 27038336 10.1113/JP272132 PMC5009781

[65] Wei S, Du H, Li Z, Tao G, Xu Z, Song X, et al. Transcription factors Sp8 and Sp9 regulate the development of caudal ganglionic eminence-derived cortical interneurons. J Comp Neurol. 2019;527:2860–74. 31070778 10.1002/cne.24712

[66] Combeau G, Kreis P, Domenichini F, Amar M, Fossier P, Rousseau V, et al. The p21-activated kinase PAK3 forms heterodimers with PAK1 in Brain Implementing Trans-regulation of PAK3 activity. J Biol Chem. 2012;287:30084–96. 22815483 10.1074/jbc.M112.355073 PMC3436265

[67] Harms FL, Kloth K, Bley A, Denecke J, Santer R, Lessel D, et al. Activating mutations in PAK1, Encoding p21-activated kinase 1, cause a neurodevelopmental disorder. Am J Hum Genet. 2018;103:579–91. 30290153 10.1016/j.ajhg.2018.09.005 PMC6174322

[68] Horn S, Au M, Basel-Salmon L, Bayrak-Toydemir P, Chapin A, Cohen L, et al. De novo variants in PAK1 lead to intellectual disability with macrocephaly and seizures. Brain. 2019;142:3351–9. 31504246 10.1093/brain/awz264 PMC6821231

[69] Ohori S, Mitsuhashi S, Ben-Haim R, Heyman E, Sengoku T, Ogata K, et al. A novel PAK1 variant causative of neurodevelopmental disorder with postnatal macrocephaly. J Hum Genet. 2020;65:481–5. 32005903 10.1038/s10038-020-0728-8

[70] Huang W, Zhou Z, Asrar S, Henkelman M, Xie W, Jia Z. p21-activated kinases 1 and 3 control brain size through coordinating neuronal complexity and synaptic properties. Mol Cell Biol. 2011;31:388–403. 21115725 10.1128/MCB.00969-10 PMC3028630

