# Supplementary material for: PAK3 activation promotes the tangential to radial migration switch of cortical interneurons by increasing leading process dynamics and disrupting cell polarity
Source: Mol Psychiatry. 2024 Mar 7;29(8):2296–307. doi: 10.1038/s41380-024-02483-y (PMC11412908; doi:10.1038/s41380-024-02483-y)
Supplement: Supplementary file 1 — Supplementary information (docx file) [file 41380_2024_2483_MOESM1_ESM.docx]

**SUPPLEMENTARY INFORMATION**

**SUPPLEMENTARY MATERIALS & METHODS**

**Mice.** Experiments have been validated and approved by the Charles Darwin Ethical committee (C2EA-05, authorized project 02241.02) and mice were housed and mated according to European guidelines. CAG-mRFP1 transgenic mice ubiquitously express the RFP (generous gift from N. Hadjantonakis^38^), Myosin 2B-GFP mutant mice express the GFP-tagged full length NMHC II-B^39^. Transgenic mice expressing Nkx2.1-cre were crossed with AI9 transgenic mice to generate Nkx2.1-cre,Rosa26-tdTomato mice that express Tomato in MGE-derived cells. Swiss female from Janvier Labs were crossed with transgenic males to produce E14.5 embryos. The day of the vaginal plug was noted E0.5.

**Construction of eGFP-PAK3 and mRFP-PAK3 plasmids.**

A polylinker (P1, P2) was inserted in the EcoR1 site of the pCAG vector (gift from Prof Miyzaki, Osaka University) to generate a pPaGi vector with unique BspEI, XhoI, ClaI, EcoRI sites. GFP-PAK3 fragments were amplified with P3, P4 primers, to add BspeI and ClaI sites to the following plasmids: pGFP-PAK3a-WT,-K297L,-T421E ^43^. Fragments were then sub-cloned in the pPaGi vector by double digestion BspeI/ClaI, to obtain the following plasmids: pPaGi-GFP-PAK3a-WT to express wild type PAK3-GFP fusion protein (eGFP-PAK3-wt); pPaGi-GFP-PAK3a-K297L to express kinase-dead mutant PAK3-GFP (eGFP-PAK3-kd); pPaGi-mRFP-PAK3a-T421E to express constitutively active mutant PAK3-GFP (eGFP-PAK3-ca).

The mRFP fragment was amplified from a mRFP-actin plasmid (Gift from Cécile Gauthier-Rouvière) with primers (P5, P6) that add HindIII and BamHI sites. It was then introduced in the pcDNA3 vector digested with HindIII/BamHI to result in the pcDNA3-mRFP plasmid. BamHI-XbaI fragments of PAK3 constructs were obtained from plasmids pcDNA3-HA-PAK3a-WT, K297L, T421E ^43^ and introduced in the pcDNA3-mRFP vector by BamHI-XbaI digestion, to obtain pcDNA3-mRFP-PAK3a-WT, K297L, and T421E.

The mRFP-PAK3 fragments were amplified with primers P7, P4 to add BspeI and ClaI sites to plasmids pcDNA3-mRFP-PAK3a-WT, K297L, and T421E. The fragments were sub-cloned in the pPaGi vector by double digestion BspeI-ClaI, to generate the following plasmids: pPaGi-mRFP-PAK3a-WT (RFP-PAK3-wt); pPaGi-mRFP-PAK3a-K297L (RFP-PAK3-kd); pPaGi-mRFP-PAK3a-T421E (RFP-PAK3-ca).

**Primers:**

P1 : 5’-AATT CTCCGGACTCGAGAATCGAT- 3’

P2 : 5’ -AATTA TCGATTCTCGAGTCCGGAG- 3’

P3 : 5’ –CGGCTCCGGAGCCACCATGGTCAGCAAGGGCGAGGA- 3’

P4 : 5’ –CGCATCGATTCTAACGGCTACTGTTC- 3’

P5 : 5’ –AAGCTTGCCACCATGGCCTCCTCCGAG- 3’

P6 : 5’ –GGATCCGTGGCGCCGGTGGAGTGGCGGCC- 3’

P7 : 5’ -CGGCTCCGGAGCCACCATGGCCTCCTCCGAGGACGT- 3’

***In utero* electroporation.**

Glass capillaries (Narishige, Japan) were pulled and calibrated for 2-3 μl plasmid injections. After DNA injection into the lateral ventricle, 5 pulses separated by 950 ms were applied at 60V for 50 ms at an angle of 30–60° from the horizontal plane (BEX LF650P2 electrodes; BTX electroporator). Embryos were left to develop *in utero* and sacrificed at E16.5. A piece of each embryo was collected for immediate PAK3 genotyping. Brains were collected in cold PBS and immediately fixed in cold 4% (wt/vol) paraformaldehyde (PFA) in 0.12 M phosphate buffer (pH 7.4) overnight. Brains were sectioned using a vibratome in the coronal plane at a thickness of 50 μm.

In each electroporated embryo, the cortical section containing the largest number of transfected cells was selected and transfected cells counted within dorsal and lateral sectors of same width. In both sectors, we moreover analyzed the distribution of cells in the embryonic cortical layers.

**Electroporation of MGE explants.**

Briefly, MGE explants were placed in a small well of 3% agar and plasmids diluted in PBS and 0.01% Fast Green were microinjected within the explant. Explants placed in a petri dish equipped with electrodes (Nepagene, Sonidel, UE) were electroporated with one pulse (100 V, 5 msec) delivered by a BTX electroporator. Electroporated explants were cultured (37°C, 5% CO2) for 5-6 hours in F12/DMEM medium with 10% calf serum.

The culture medium contained 0.4% glucose, 1% penicillin/streptomycin, HEPES 10mM in glutamax supplemented F12-DMEM, all products from Gibco.

**Culture of forebrain slices.** 300-μm thick coronal sections of the forebrain of E14.5 Nkx2.1-cre:dtTomato transgenic embryos were prepared using a vibratome. Sections were cultured for 24 to 30 hours in Millicell chambers using culture medium supplemented with 10% fetal calf serum, which contained either the vehicle (DMSO 1/10000), or 1 μM FRAX597 (stock solution 10mM FRAX597 in DMSO). After fixation in 4% PAF 0.12M Phosphate buffer, slices were processed for bisbenzimide staining and mounted in glycerol. Sectors were imaged with a confocal microscope (x40 objective) in the lateral and medial cortex. Pictures were acquired every 2 μm in a total thickness of 40 μm at a distance from the sectioned surface to avoid border effects.

**Immunohisto- and cyto-chemistry.** Embryonic brains were fixed overnight in 4% PFA in 0.12M Phosphate Buffer at 4°C, rinsed, embedded in agarose 3% and sectioned with a vibratome at 60 μm. Embryonic brains sectioned at 14 μm with a cryostat were cryoprotected in PBS 10% sucrose after fixation in 4% PFA. Organotypic slices were fixed by immersion in cold 4% PFA. Co-cultures were fixed at least 3 hours in 4% PFA/ 0.33 M sucrose in 0.12M Phosphate Buffer at 4°C for GFP staining.

Cultures were preincubated for two hours in PBT (PBS; 0.25% Triton X-100) with 10% normal goat serum. Organotypic brain slices grafted with electroporated explants were pre-incubated in PGT (PBS; gelatin 2g/L; 0,25% (wt/vol) Triton X-100) for more than 5 hours before GFP amplification and brain sections were preincubated for two hours in PGT. Tissues or cells were then incubated overnight at 4°C in primary antibodies respectively diluted in PGT or PBT with 2% normal goat serum and 1% bovine serum albumin (BSA, Sigma-Aldrich). Primary antibodies were chicken anti-GFP (1:5000 Aves Lab), rabbit anti-GFP serum (1:1000 Invitrogen), rabbit anti-RFP (1:1000, BioVision), rabbit anti-Active Caspase-3 (1:200, BD Biosciences, clone C92-605), mouse anti-PAK3 (1/100, Sigma-Aldrich, clone 3A12), rabbit anti-phosphorylated PAK3 (phospho Ser154) (1:100, antibodies.com, A94421). After rinses with PBT, sections were incubated 1h30 with appropriate secondary antibodies (Alexa dye (Molecular Probes) or Cy3 (Jackson laboratories) conjugated) diluted in PBT at 1/400. Brain sections were extensively washed in PBS after antibody incubation. Bisbenzimide (1/5000 in PBT, Sigma) was used for nuclear counterstaining. F-actin was detected with phalloidin coupled to alexa Fluor (1/40 in PBT, 20 minutes, Invitrogen). Brain sections and cultures were mounted in Mowiol-Dabco, organotypic slices in glycerol/PBS (2/1 vol/vol).

***In situ* Hybridization**

*In situ* Hybridization was performed following the protocol described in^40^ on 100 μm floating sections prepared with a vibratome

**SUPPLEMENTARY FIGURES**

**
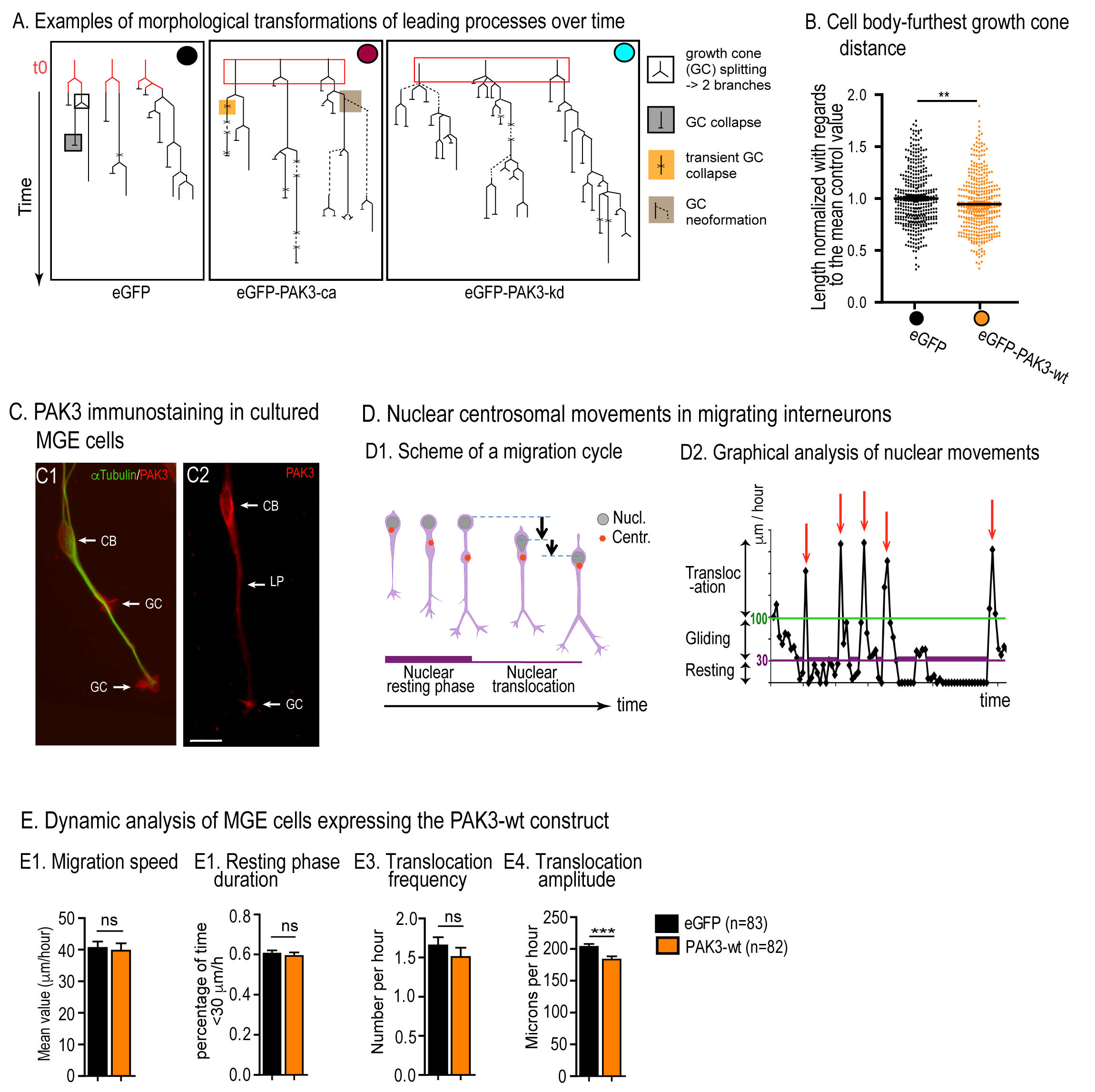
Supplementary Figure S1 (related to Figures 1 and 2):**

**Leading process transformation and nuclear movements in transfected MGE cells .**

**A**. Dendograms schematize the leading process transformations of MGE cells electroporated with eGFP (black dot), eGFP-PAK3-ca (red dot), and eGFP-PAK-kd (blue dot) constructs over time. Four main growth cone transformations indicated in the legend were identified and their frequency calculated (see histograms G in Fig.1). See also supplementary MovieS1A. **B.** Measure of the longest neurite in leading processes of MGE cells migrating away from their explant of origin on dissociated cortical cells. Comparisons between MGE cells electroporated with eGFP (black) and eGFP-PAK3-wt (orange) plasmids. Statistical significance was assessed by Mann Whitney tests (** p=0.006). **C**. Panels C1 and C2 show that PAK3 antibodies strongly label the leading growth cone (GC), cell body (CB) and proximal part of the leading process (LP) of MGE cells in culture. Anti α-Tubulin antibodies in C1 label microtubules. Scale bar, 10 μm. **D1-D2.** The migration cycle (D1) of cortical INs shows two main phases: 1) forward centrosomal migration during a nuclear resting phase, 2) nucleus translocation towards the centrosome. Graph in D2 defines the 3 modes of nuclear movements analyzed in the present study: i) resting phases for instantaneous migration speeds <30 microns per hour, ii) gliding movements for instantaneous migration speeds between 30 and 100 microns per hour iii) nuclear translocations (red arrows) for instantaneous migration speeds >100 microns per hour. **E.** Histograms compare the mean migration speed (E1), the duration of resting phases (E2), the frequency (E3) and amplitude (E4) of nuclear translocations in MGE cells expressing eGFP (black histogram bars) and eGFP-PAK3-wt (orange histogram bars). Statistical significance was assessed by Mann-Withney tests (*** p=0.0002).

**
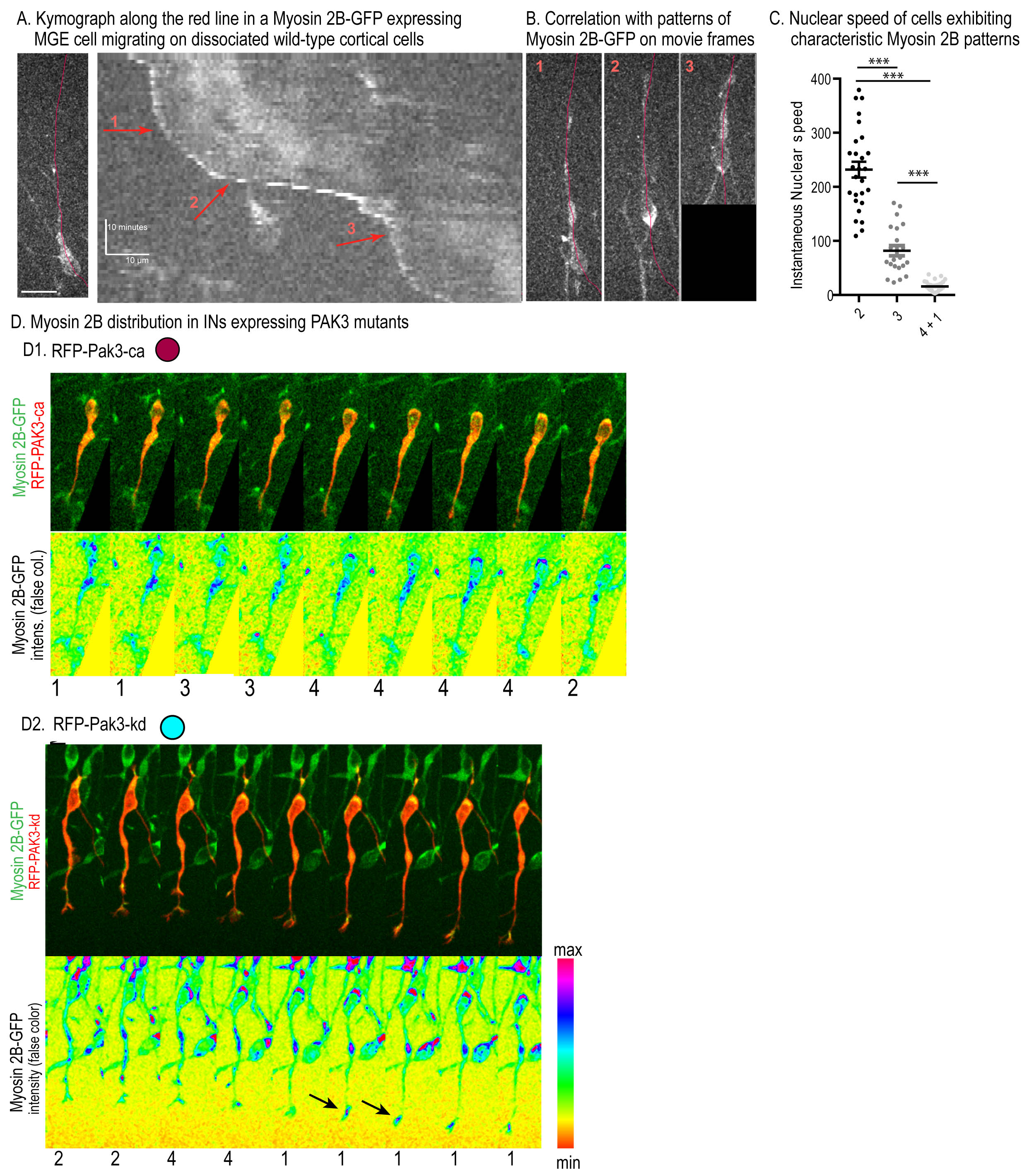
Supplementary Figure S2 (related to Fig.2):**

**Patterns of myosin 2B distribution during the migration cycle in MGE cells.**

**A.** Kymograph shows the temporal evolution along the red line (picture on the left) of the GFP signal in a MGE cell expressing a myosin 2B-GFP during migration. Scale bars on kymographs show time (y=10 minutes) and distance (x= 10 microns). **B.** Illustration of myosin 2B-GFP distributions corresponding to the patterns 1,2,3 defined in Fig.2G and identified on the kymograph. **C.** Histogram of cell rear migration speeds calculated on kymographs of 11 migrating cells and expressed as a function of the myosin 2B-GFP patterns defined in Fig.2G. Fast nuclear movements were correlated with a strong myosin 2B-GFP signal at the nuclear rear (pattern 2), whereas intermediate nuclear speeds were measured when myosin 2B-GFP relocated at the nuclear front (pattern 3). Statistical significance was assessed by One Way ANOVA (p<0.0001) and Tukey’s multiple comparison tests. **D.** Time-lapse sequences illustrate the distribution of the GFP signal in myosin 2B-GFP MGE cells electroporated with RFP-PAK3-ca (D1, see related MovieS2D1) or RFP-PAK3-kd (D2, see related MovieS2D2) constructs. The bottom row in each sequence shows the GFP intensity in false colors (scale on the bottom right). Numbers under frames refer to the myosin 2B-GFP pattern (see definition in Fig.2F,G).

**
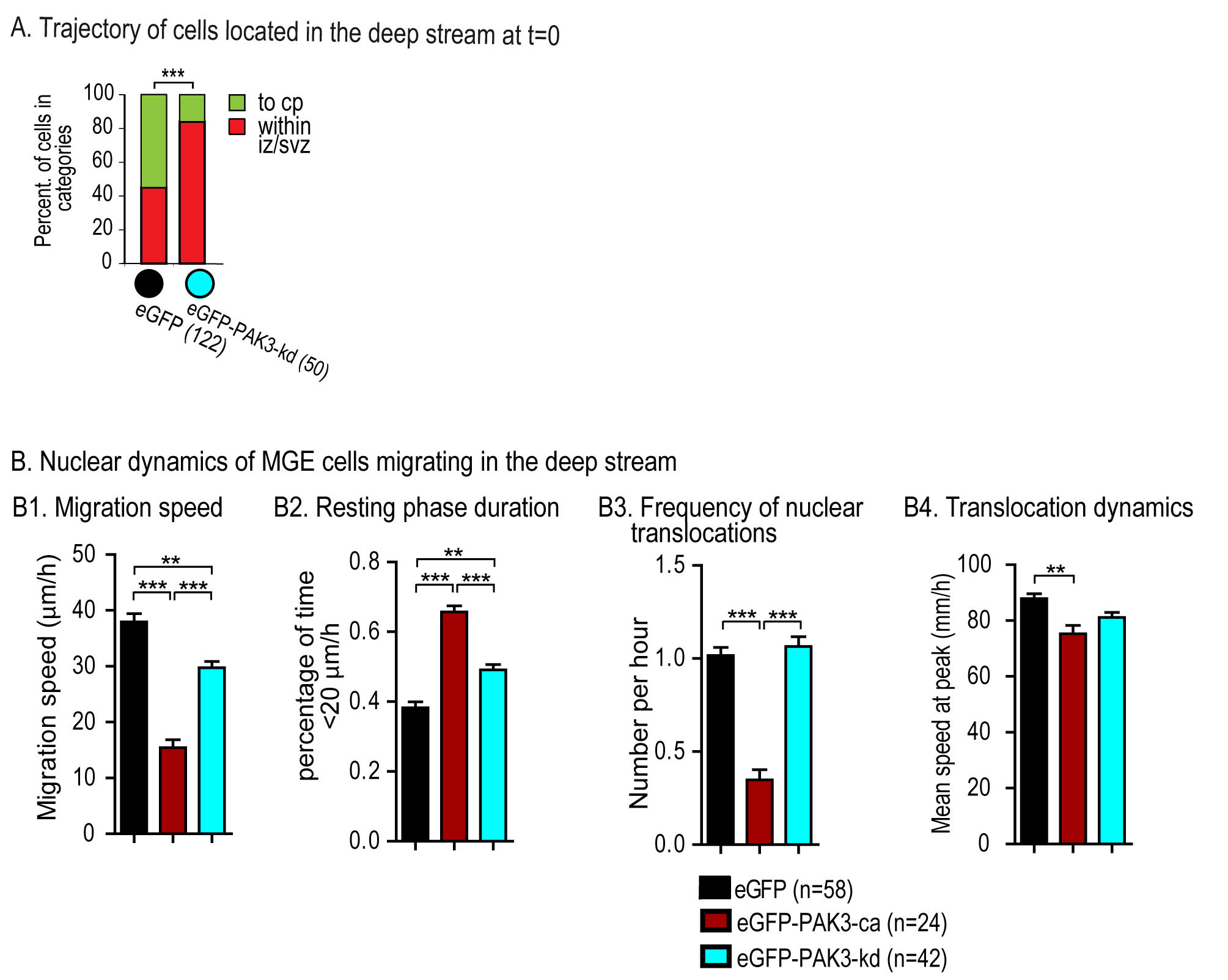
 Supplementary Figure S3 (related to Fig.3 and 4):**

**Trajectory and dynamics of electroporated MGE cells grafted in cortical slices.**

**A.** Histogram compares the frequency of trajectories remaining in the iz/svz (red) and reoriented to the cp (green) for MGE cells expressing eGFP and PAK3-kd and located in the deep migration stream at the start of the recording session (chi2 tests, p<0.0001). **B.** Histograms compare the mean migration speed (B1), the total duration of resting phases (B2), the frequency of nuclear translocations (B3) and the mean migration speed at peaks (B4) of MGE cells expressing eGFP (black histogram bars, 3 cortical slices), eGFP-PAK3-ca (red histogram bars, 2 cortical slices) and eGFP-PAK3-kd (blue histogram bars, 2 cortical slices) and grafted in cortical slices imaged every 10 minutes for more then 20 hours. Migrating MGE cells were imaged in the deep stream (red trajectories in Fig.3F). The threshold speed to define nuclear translocations was 50 μm/hour. Statistical significance was assessed by Kruskal-Wallis tests (p<0.0001 for all comparisons) and Dunn’s multiple comparison tests.

**
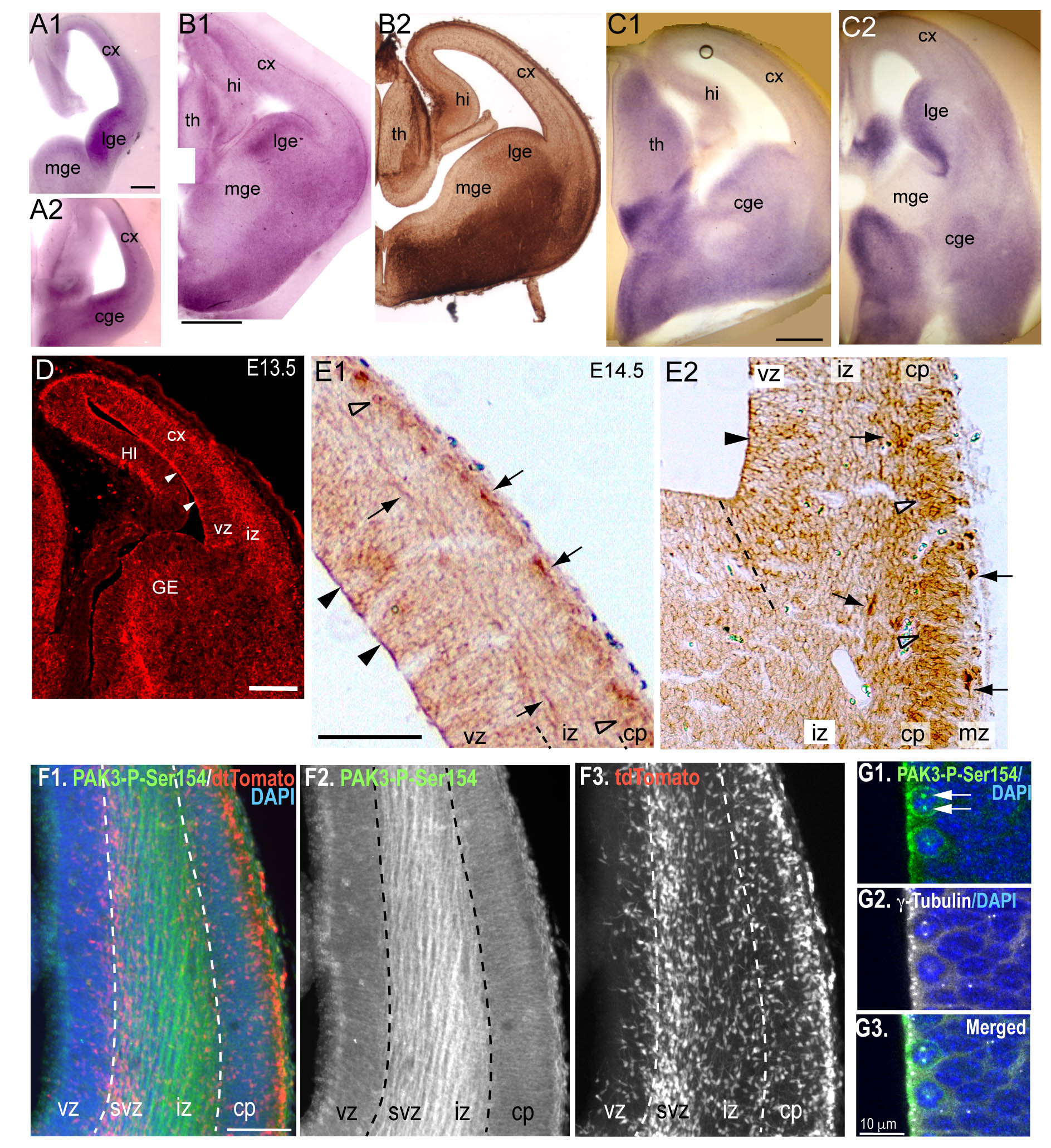
 Supplementary Figure S4 (related to Figure 5): *Pak3* expression in the mouse forebrain at embryonic stages E12.5 - E14.5 and cortical expression of PAK3 at E13.5 - E14.5.**

**A1-C2**: In situ hybridization with an anti-Pak3 probe of embryonic forebrain sections at E12.5 (medial -A1- and caudal -A2- frontal sections), E13.5 (B1- medial frontal section) and E14.5 (-C1- caudal frontal section, -C2- horizontal section). At all stages, a strong signal is observed in the vz of the lateral (LGE) and caudal (CGE) ganglionic eminences. The proliferative zones of the medial ganglionic eminence (MGE) appear free of signal. Panel B2 shows the distribution of GABA immunopositive cells on a frontal forebrain section at E13.5, for comparison with B1. **D-E2**. PAK3 immunostaining of frontal cryostat sections of wild type embryos at E13.5.(D) and E14.5 (E1-E2). D. Immunopositive cells are numerous in the svz of the GE, in the iz of the developing cx, at the pial surface of the HI, and on the ventricular surface of the cx (white arrows). E1-E2 are enlarged views of the dorsal (E1) and lateral (E2) cortex showing tangentially oriented PAK3 immunopositive(+) cells in the mz and iz, and radially oriented PAK3(+) cells in the vz. Faint staining is observed in the cp. **F1-G3.** Immunostaining with anti-activated PAK3-P-Ser154 antibodies of frontal cortical sections from E14.5 transgenic embryos expressing dtTomato in MGE cells (Nkx2.1-cre, Ai9 mouse strain). PAK3-P-Ser154 antibodies strongly label tangentially oriented structures (F1,F2) in the iz and svz, where Tomato-positive MGE-derived cells migrate tangentially. Enlarged views of cortical progenitors located along the ventricle (G1-G3) show that PAK3-P-Ser154 antibodies label the γ-tubulin positive centrioles (G2,G3) and the cellular cortex (G1,G3) of a subset of progenitors. cx, cortex; hi, hippocampus; th, thalamus; vz, ventricular zone; svz, subventricular zone; iz, intermediate zone; sp, subplate; cp, cortical plate; mz, marginal zone. . Scale bars: A1, 200 μm; B1,C1, 500 μm; D, 200 μm; E1, 100 μm ; F1, 100 μm, G3, 10 μm.

**
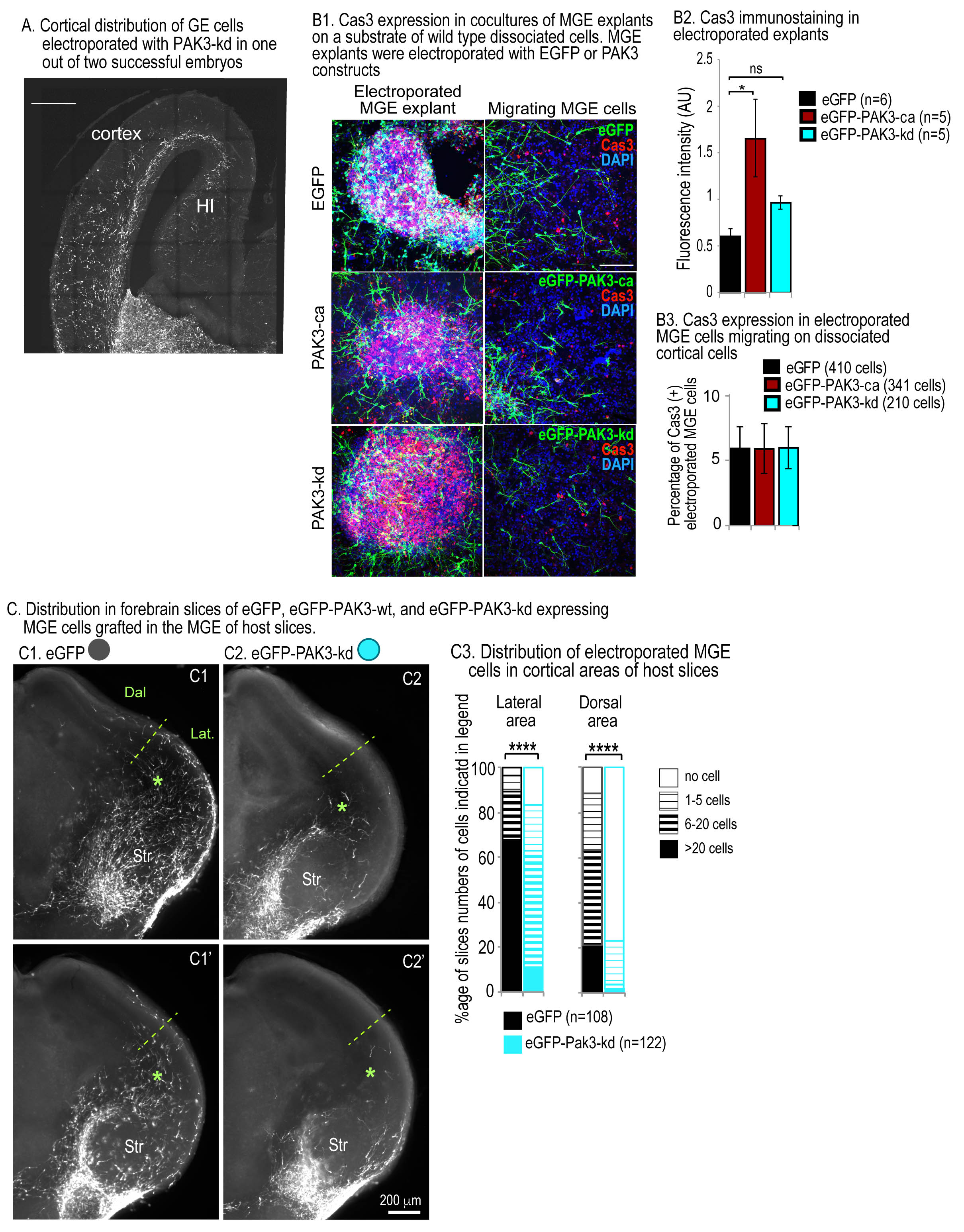
Supplementary Figure S5 (related to Figure 5 and to Table1):**

**A.** Example of cortical section (50μm-thick, coronal) showing GE cells electroporated at E12.5 with the eGFP-PAK3-kd construct migrating at E16.5 in the cortex. This dense migratory stream was very rarely observed in the cortex (See Table 1). HI, hippocampus. Scale bar: 300 µm.

**B1-B3.** Influence of the electroporation of PAK3 constructs on the expression of active caspase-3 in MGE cells. B1. Confocal pictures show at the same magnification electroporated MGE explants (left column, z-projection over 20 μm) and transfected cells that left the MGE explants and migrated on the dissociated cortical cells (right column, z-projection over 10 μm) in cocultures. Immunostaining shows eGFP (green) and Active-Caspase-3 (red) expression. Scale bar, 50 μm. B2. Active Cas-3 immunostaining was evaluated in electroporated MGE explants by measuring the mean fluorescence intensity in 30 confocal planes of the explant. It was increased in explants electroporated with PAK3-ca (One-way ANOVA, and Dunn’s post-hoc tests, *,p=0.022). B3. Whatever the electroporated construct, the same low proportion of migrating MGE cells expressed active Cas-3 immunostaining. **C.** **C1-C2** and **C’1-C’2** are representative pictures of wild type forebrain slices grafted at E14.5 in the MGE with MGE explants electroporated with either eGFP (C1, C’1) or PAK3-kd (C2, C’2) constructs, fixed at E16.5, immunostained with anti-GFP antibodies and mounted in glycerol. Migratory streams observed in the basal forebrain most often reached the lateral cortex. In histogram C3, the numbers of grafted MGE cells located within the lateral and dorsal cortical regions, respectively below and above the dotted line, were classified as explained in legend. The green star indicates the ventricular angle at the pallium-subpallium boundary. The dotted line is 200 μm dorsal to the ventricular angle. Scale bar, 200 μm.

**LEGENDS OF SUPPLEMENTARY MOVIES**

**Movie 2E (related to Fig.2E): Migration of a Myosin 2B-GFP expressing MGE cell electroporated with mRFP**. A MGE explant from a Myosin 2B-GFP embryo has been electroporated and cultured on wild-type dissociated cortical cells. Time-lapse between frames is 1 minute (sequence of 1.5 hour, 10 frames per second).

**Movie 3D1 (related to Fig.3D1): Migration of MGE cells expressing eGFP in an organotypic cortical slice.** The electroporated MGE explant has been grafted in the lateral cortex. EGFP(+) cells migrate away from the explant toward the medial cortex and colonize all cortical layers. Time-lapse between frames is 10 minutes (sequence of 10 hours, 15 frames per second).

**Movie 3D2 (related to Fig.3D2): Migration of MGE cells expressing eGFP-PAK3-ca in an organotypic cortical slice.** The electroporated MGE explant has been grafted in the lateral cortex. EGFP(+) cells migrate away from the explant either within the cortical plate above the graft, or within a deep stream toward the medial cortex. Time-lapse between frames is 10 minutes (sequence of 10 hours, 15 frames per second).

**Movie 3D3 (related to Fig.3D3) : Migration of MGE cells expressing eGFP-PAK3-kd in an organotypic cortical slice.** The electroporated MGE explant has been grafted in the lateral cortex. EGFP(+) cells migrate away from the explant toward the medial cortex and colonize all cortical layers except the cortical plate. Time-lapse between frames is 10 minutes (sequence of 10 hours, 15 frames per second).

**MovieS1A (related to supplementary Fig.S1A): Dynamic transformations of growth cones at the end of neurite branches over time.** Dendrogram (scheme on the right side) reproduces the dynamic transformations of growth cones at the end of neurite branches over time, as they appear on the time-lapse movie of a migrating interneuron (movie on the left side). Arrowheads are migrating growth cones, bars represent growth cone collapse, and yellow branches are growth cone splitting. Time-lapse between frames is 3 minutes (sequence of 1.0 hour, 10 frames per second).

**MovieS2D1 (related to supplementary Fig.S2D1): Migration of a Myosin 2B-GFP expressing MGE cell electroporated with mRFP-PAK3-ca**. An MGE explant from a Myosin 2B-GFP embryo has been electroporated and cultured on wild-type dissociated cortical cells. Time-lapse between frames is 1 minute (sequence of 1.85 hour, 10 frames per second).

**MovieS2D2 (related to supplementary Fig.S2D2): Migration of a Myosin 2B-GFP expressing MGE cell electroporated with mRFP-PAK3-kd**. An MGE explant from a Myosin 2B-GFP embryo has been electroporated and cultured on wild-type dissociated cortical cells. Time-lapse between frames is 1 minute (sequence of 1.8 hour, 10 frames per second).

**Number of replicates**

**Figure 1**

1D, E: 3 eGFP, 3 PAK-kd and 4 PAK3-ca co-cultures in 3 independent experiments. Total number of cells shown in legend 1D.

1F, 1G: 2 independent recording experiments of control and PAK3 mutant cells.

1F: 4 movies of eGFP-expressing cells, 3 movies of PAK3-ca expressing cells, 3 movies of PAK3-kd expressing cells. Number of analyzed growth cones shown in legend (at least 25 cells per condition).

1G. 5 movies of eGFP-expressing cells (19 cells), 5 movies of PAK3-ca expressing cells (18 cells), 5 movies of PAK3-kd expressing cells (12 cells). Cells analyzed for 2h30 to 5h00 hours (50 to 100 frames).

**Figure 2**

2B: 5 independent recording experiments of control and PAK3 mutant cells electroporated with the PACT-mKO1 construct. 12 movies of control cells (65 cells), 7 movies of cells expressing PAK3-ca (35 cells), 6 movies of cells expressing PAK3-kd (49 cells).

2C,D1,D2: 3 independent recording experiments of control and PAK3 mutant cells. 11 movies of eGFP expressing cells (133 cells), 8 movies of PAK3-ca expressing cells (94 cells), 6 movies of PAK3-kd expressing cells (85 cells).

2F: Analysis of 13 control cells expressing the myosin 2B-GFP fusion protein

2G: 4 independent recording experiments. 6 movies of myosin 2B-GFP expressing cells electroporated with eGFP (28 cells), 4 movies of myosin 2B-GFP expressing cells electroporated with PAK3-ca (14 cells), 3 movies of myosin 2B-GFP expressing cells electroporated with PAK3-kd (13 cells).

**Figure 3**

3A, B, C: 5 independent recording experiments. 17 movies of control cells expressing eGFP, 9 movies of cells expressing PAK3-ca, 11 movies of cells expressing PAK3-kd. Numbers of cells indicated in legend.

3D: 7 independent recording experiments of slices grafted with a MGE explant: 3 explants expressing eGFP (tracking of 132 cells), 2 explants expressing PAK3-ca (tracking of 29 cells) expressing PAK3-kd (tracking of 67 cells).

**Figure 4**

4A,B,C: 9 repeated experiments. 49 slices (8 brains) grafted with MGE explants expressing eGFP, 35 slices (7 brains) grafted with MGE explants expressing PAK3-ca, 45 slices (7 brains) grafted with MGE explants expressing PAK3-kd.

4D: 2 repeated experiments. 15 slices (6 brains) grafted with eGFP expressing MGE explants (8 cultured with vehicle, 7 with FRAX597); 16 slices (6 brains) grafted with PAK3-ca expressing MGE explants (8 cultured with vehicle, 8 with FRAX597).

**Figure 5**

A: 3 independent experiments. Immunostaining of 4 brains from 3 litters of E14.5 Nkx2.1-cre,Rosa26-tdTomato embryos.

B,C,D: 4 brains with electroporated GE cells in the cortex for each construct (eGFP, PAK3-wt, PAK3-ca). Section with the highest number of GFP expressing cells in each brain.

C: 328 cells expressing eGFP, 152 cells expressing PAK3-wt, 93 cells expressing PAK3-ca (dorsal sector). D: 360 cells expressing eGFP, 319 cells expressing PAK3-ca.

E: 2 independent culture experiments. Sections from 4 control brains (Nkx2.1-cre; AI9 + vehicle) and 4 treated brains (Nkx2.1-cre; AI9 + FRAX597). Analysis of 1 section per brain.

**Supplementary Figure S1**

B: 3 eGFP and 3 PAK-wt independent co-cultures. 397 control eGFP-expressing cells, 372 PAK3-wt expressing cells.

C: immunostaining of 4 independent coverslips (4 or 5 MGE explants per coverslip)

E. 2 independent recording sessions, 6 movies of PAK3-wt expressing MGE cells (82 cells), 5 movies of eGFP-expressing MGE cells (83 cells).

**Supplementary Figure S2**

C: 10 kymographs in 2 independent recording experiments. 10 movies.

**Supplementary Figure S3**

Slices grafted with a MGE explant. 7 independent recording experiments: 3 eGFP expressing explants, 2 PAK3-ca expressing explants, 2 PAK3-kd expressing explants.

A: Number of analyzed cell trajectories shown in legend.

B: Tracking and analysis of cells migrating in the deep stream. Number of cells indicated in legend.

**Supplementary Figure S4**

A1-C2: In situ hybridization repeated 3 times or more at each stage (E12.5, E135, E14.5).

D,E: experiments repeated more than 3 times

F,G: Immunostaining of 4 brains from 3 litters of E14.5 Nkx2.1-cre,Rosa26-tdTomato embryos.

**Supplementary Figure S5**

B1-B3: 2 sets of experiments. 6 eGFP expressing MGE explants, 5 PAK3-ca expressing explants, 5 PAK3-kd expressing explants.

C. 4 independent grafting experiments. 54 slices from 16 brains grafted bilaterally with eGFP expressing MGE explants; 61 slices from 28 brains grafted bilaterally with PAK3-kd expressing MGE explants
